# Enzymatic reactions of AGO4 in RNA-directed DNA methylation: siRNA duplex loading, passenger strand elimination, target RNA slicing, and sliced target retention

**DOI:** 10.1101/gad.350240.122

**Published:** 2023-02-01

**Authors:** Feng Wang, Hsiao-Yun Huang, Jie Huang, Jasleen Singh, Craig S. Pikaard

**Affiliations:** 1 Department of Biology, Indiana University, Bloomington, Indiana 47405, USA;; 2 Department of Molecular and Cellular Biochemistry, Indiana University, Bloomington, Indiana 47405, USA;; 3 Howard Hughes Medical Institute, Indiana University, Bloomington, Indiana 47405, USA

**Keywords:** ARGONAUTE, short interfering RNA, noncoding RNA, transcriptional gene silencing, chromatin modification, RNA polymerase V

## Abstract

RNA-directed DNA methylation in plants is guided by 24-nt siRNAs generated in parallel with 23-nt RNAs of unknown function. We show that 23-nt RNAs function as passenger strands during 24-nt siRNA incorporation into AGO4. The 23-nt RNAs are then sliced into 11- and 12-nt fragments, with 12-nt fragments remaining associated with AGO4. Slicing recapitulated with recombinant AGO4 and synthetic RNAs reveals that siRNAs of 21–24 nt, with any 5′-terminal nucleotide, can guide slicing, with sliced RNAs then retained by AGO4. In vivo, RdDM target locus RNAs that copurify with AGO4 also display a sequence signature of slicing. Comparing plants expressing slicing-competent versus slicing-defective AGO4 shows that slicing elevates cytosine methylation levels at virtually all RdDM loci. We propose that siRNA passenger strand elimination and AGO4 tethering to sliced target RNAs are distinct modes by which AGO4 slicing enhances RNA-directed DNA methylation.

## Abstract (precis)

In this study, Wang et al. investigated the function of 23-nt RNAs in plants, which are generated in parallel with 24-nt siRNAs that guide RNA-directed DNA methylation. They show that 23-nt RNAs function as passenger strands during 24-nt siRNA incorporation into AGO4, and propose that siRNA passenger strand elimination and AGO4 tethering to sliced target RNAs are distinct modes by which AGO4 slicing enhances RNA-directed DNA methylation.

In eukaryotes, RNA silencing plays a significant role in gene regulation, transposable element repression, and genome stability. A common theme among RNA silencing pathways is that small RNAs stably associate with Argonaute family proteins and then interact with complementary target RNAs to interfere with RNA translation or to bring about chromatin modifications that repress gene transcription (Martienssen and Moazed 2015; Iwakawa and Tomari 2022). An ancestral and fundamental function shared by many Argonaute proteins is small RNA-programmed endonucleolytic slicing of the targeted RNA at a phosphodiester bond (Joshua-Tor and Hannon 2011).

RNA-directed DNA methylation (RdDM) is the major transcriptional gene silencing pathway in plants (Matzke and Mosher 2014; Zhou and Law 2015; Wendte and Pikaard 2017; Zhang et al. 2018). RdDM siRNA precursors are synthesized by DNA-dependent nuclear RNA polymerase IV (Pol IV) (Ream et al. 2009; Haag and Pikaard 2011) and RNA-dependent RNA polymerase 2 (RDR2) (Zhou and Law 2015; Fukudome et al. 2021; Du et al. 2022). These enzymes physically associate and carry out coupled reactions that transcribe target locus DNA sequences into double-stranded RNAs of ∼25–40 bp (Haag et al. 2012; Singh et al. 2019; Fukudome et al. 2021; Huang et al. 2021). DICER-LIKE 3 (DCL3) then cuts the dsRNAs into siRNAs, guided by sequence and structural features at the ends of precursor duplexes (Loffer et al. 2022). The resulting dicing products consist of a 24-nt siRNA strand paired with either a 23-nt strand (24/23 duplexes) or a 24-nt strand (24/24 duplexes) (Nagano et al. 2014; Loffer et al. 2022), one strand of which ultimately becomes stably associated with ARGONAUTE 4 (AGO4). The siRNAs then guide AGO4 to RdDM target loci by base-pairing to transcripts generated by nuclear RNA polymerase V (Pol V) (Wierzbicki et al. 2008, 2009; Liu et al. 2018), with protein–protein interactions between AGO4 and the Pol V largest subunit (NRPE1) and/or the Pol V-associated protein SPT5L also implicated in target locus interactions (Li et al. 2006; El-Shami et al. 2007; Bies-Etheve et al. 2009). The resulting AGO4–siRNA–Pol V complexes enable recruitment of the de novo cytosine methyltransferase DRM2 (Zhong et al. 2014; Fang et al. 2021) as well as enzymes that reposition or chemically modify the histone proteins that wrap the DNA (Du et al. 2015). Collectively, these activities bring about chromatin environments that repress promoter-dependent gene transcription by RNA polymerases I, II, or III (Fig. 1A; Preuss et al. 2008; Blevins et al. 2009).

**Figure 1.**
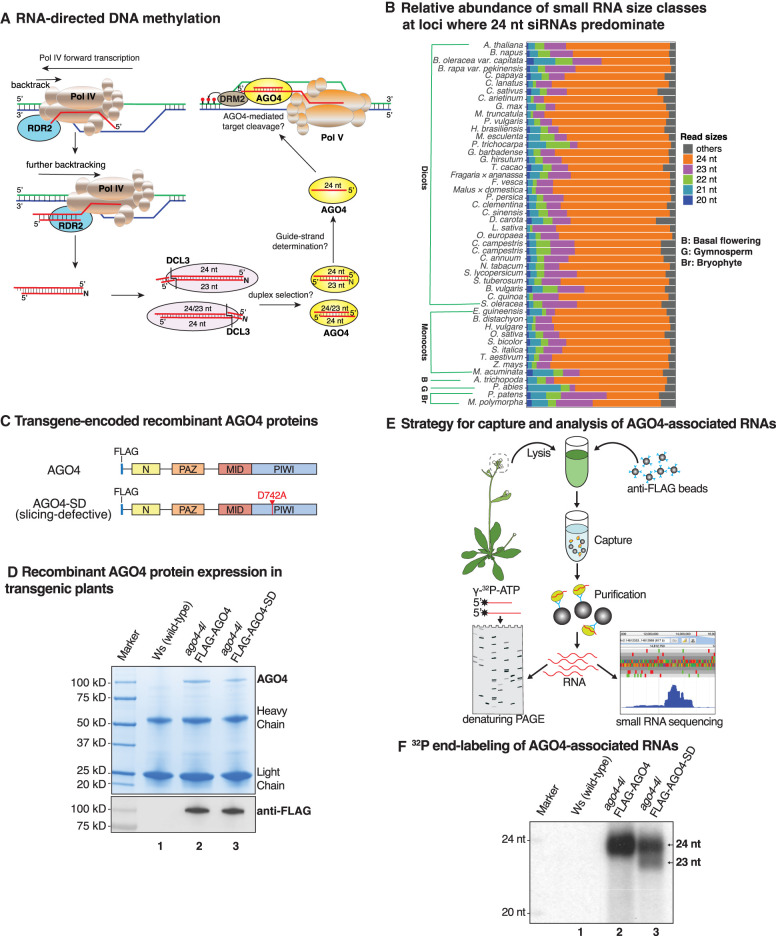
Evidence that 23-nt siRNAs function as passenger strands for 24-nt guide RNAs. (*A*) A simplified model for RNA-directed DNA methylation highlighting questions addressed in our study. (*B*) Relative abundance of small RNA size classes at 24-nt siRNA-dominated loci in diverse species (Lunardon et al. 2020). (*C*) Transgene-encoded AGO4 proteins expressed from the native promoter in *ago4-4*-null mutant plants (ecotype Ws). (*D*) Comparison of AGO4 and AGO4-SD levels immunoprecipitated from inflorescence tissue. Duplicate gels were stained with Coomassie brilliant blue (*top* panel) or subjected to immunoblotting using anti-FLAG antibody (*bottom* panel). Nontransgenic Ws served as a control in lane *1*. (*E*) Cartoon depicting AGO4 affinity capture and analysis of associated RNAs by ^32^P end-labeling or deep sequencing. (*F*) Autoradiograph of 5′ end-labeled RNAs in anti-FLAG IP fractions from nontransgenic plants (lane *1*), plants expressing FLAG-AGO4 (lane *2*), or plants expressing FLAG-AGO4-SD (lane *3*).

Although DCL3 dicing frequently generates duplexes consisting of 23-nt siRNAs base-paired with 24-nt siRNAs (Loffer et al. 2022), siRNAs that coimmunoprecipitate with AGO4 are almost exclusively 24 nt (Qi et al. 2006; Havecker et al. 2010; Wang et al. 2011). Thus, the fates and functions of 23-nt siRNAs are unclear. Using genetic and genomic approaches, as well as enzymatic reactions catalyzed by recombinant AGO4–siRNA complexes reconstituted in vitro, we show that 23-nt siRNAs serve as passenger strands that specify that the 24-nt RNAs to which they are base-paired become stably associated with AGO4 as guide strands. The 23-nt RNAs are then sliced by AGO4 into 11- and 12-nt fragments. We show that AGO4 loading and slicing activity is similar for guide RNAs with A, U, C, or G at their 5′ termini and for guide RNAs that vary in length from 21 to 24 nt, indicating that the 24-nt length and 5′ A bias among AGO4-associated siRNAs in vivo are not due to AGO4 binding requirements. Surprisingly, we found that AGO4 retains fragments of the passenger strand and target RNAs that it slices, both in vitro and in vivo, suggesting a function for the retained RNAs. We propose that AGO4 slicing of target RNAs synthesized by Pol V elongation complexes causes the successive release of RNA fragments that remain locally associated with RdDM loci. By remaining bound to these sliced RNAs, multiple independent AGO4–RNA complexes could linger at the loci and act in parallel to achieve maximal levels of methylation.

## Results

### Plant 23-nt RNAs function as passenger strands for 24-nt guide RNAs

DCL3 dicing of double-stranded Pol IV-RDR2 transcripts yields duplexes with two 24-nt siRNAs (24/24 duplexes) or a 24-nt siRNA paired with a 23-nt siRNA (24/23 duplexes) (Fig. 1A; Loffer et al. 2022). Thus, at loci where 24-nt siRNAs are the predominant class of small RNA, in diverse plant species, 23-nt siRNAs are second in abundance (Fig. 1B). Small RNAs of 21 and 22 nt are also produced at these loci, ostensibly diced by DCL1, DCL2, or DCL4, and could potentially facilitate post-transcriptional silencing and/or initial low-level DNA methylation events that enable Pol IV and Pol V recruitment, thus jump-starting the major RdDM pathway diagrammed in Figure 1A (McCue et al. 2015; Hung and Slotkin 2021; Sigman et al. 2021).

Insight into the function of 23-nt siRNAs came from an experiment in which we transformed an *ago4-4*-null mutant of the *Arabidopsis thaliana* ecotype (natural strain) Wassilewskija (Ws) with transgenes expressing wild-type AGO4 or slicing-defective AGO4 (AGO4-SD), each bearing a FLAG epitope tag (Fig. 1C). In AGO4-SD, aspartate D742 is changed to alanine within the catalytic center conserved among slicing-competent Argonaute family proteins (Rivas et al. 2005). The recombinant AGO4 and AGO4-SD proteins were expressed at similar levels, as shown by anti-FLAG immunoblot detection of the proteins in total cell lysates (Supplemental Fig. S1A) and Coomassie blue staining and anti-FLAG immunoblot detection following their affinity capture using anti-FLAG antibodies (Fig. 1D). RNAs copurifying with affinity-captured AGO4 or AGO4-SD were then 5′ end-labeled with ^32^P and resolved by denaturing polyacrylamide gel electrophoresis (PAGE) or subjected to high-throughput small RNA sequencing (Fig. 1E). End labeling showed that small RNAs associated with wild-type AGO4 are almost exclusively 24 nt (Fig. 1F, lane 2), consistent with prior studies (Qi et al. 2006; Havecker et al. 2010; Wang et al. 2011). However, siRNAs associated with AGO4-SD included both 24- and 23-nt RNAs (Fig. 1F, lane 3) at relative levels consistent with their biogenesis (Singh et al. 2019; Loffer et al. 2022). These results suggest that AGO4 is initially loaded with siRNA duplexes, with one strand (the passenger strand) then eliminated by slicing (Ye et al. 2012). For 24/23 duplexes, the 23-nt strand is selectively eliminated.

### AGO4-associated small RNAs have distinct sequence features that allow interpretation of their origins

**Figure 2.**
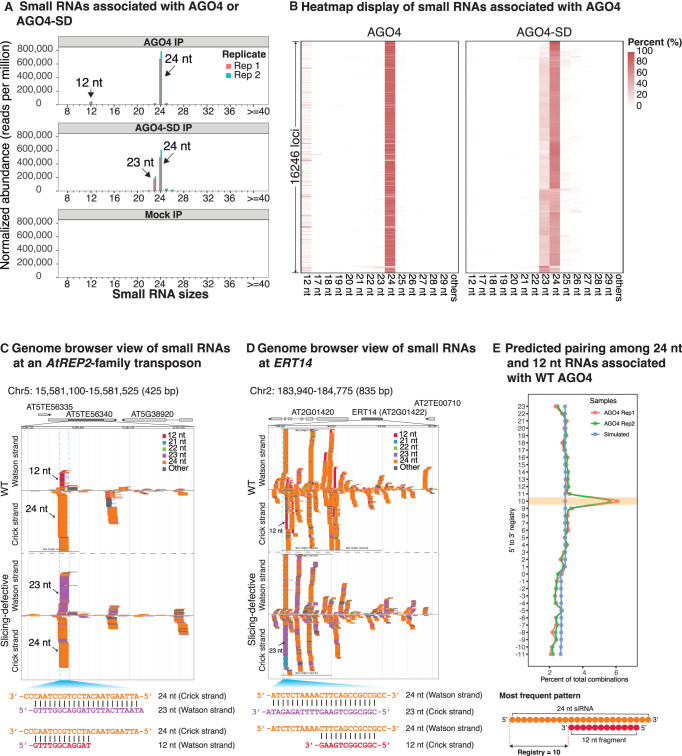
Distinct small RNA populations are associated with wild-type versus slicing-defective AGO4. (*A*) Relative abundance of small RNAs associated with AGO4 or AGO4-SD. See also Supplemental Figures S1 and S2. (*B*) Relative abundance of AGO4- or AGO4-SD-associated RNAs of different size classes at 16,246 loci where 23- or 24-nt siRNAs are the dominant size class. (*C*,*D*) Genome browser views of small RNAs copurifying with AGO4 or AGO4-SD. (*C*) *At5TE56340* is an *AtREP2* family transposon. (*D*) *ERT14* is an RdDM locus characterized by Blevins et al. (2014). RNAs are color-coded by size and are displayed *above* or *below* the central horizontal axis according to strandedness. RNAs within the regions denoted by dashed lines were used for sequence alignments at the *bottom* of *C* and *D*. See also Supplemental Figure S3. (*E*) Frequencies of various pairing registries for 12- and 24-nt RNAs associated with wild-type AGO4. The most highly represented pairing pattern is illustrated at the *bottom* of the panel. For 24- and 12-nt RNAs that overlap with at least 1 nt of complementarity, the 5′ to 3′ registry is defined as the distance from the 5′ nucleotide of the top strand to the 3′ nucleotide of the bottom strand. See Supplemental Figure S3 and the Supplemental Material for additional details.

Deep sequencing of small RNAs that copurify with FLAG-tagged wild-type AGO4 upon anti-FLAG immunoprecipitation (IP) revealed abundant 24-nt RNAs and minor classes of 12-, 23-, and 25-nt RNAs (Fig. 2A; Supplemental Fig. S1B). In contrast, RNAs associated with AGO4-SD included abundant 24- and 23-nt RNAs (Fig. 2A; Supplemental Fig. S1B) and low levels of 25- and 26-nt RNAs but no 12-nt RNAs. Nontransgenic plant samples subjected to anti-FLAG immunoprecipitation as a negative control yielded no RNA signals above background (Fig. 2A, bottom panel, labeled mock IP).

Analysis of total small RNA abundance without enrichment by AGO4 immunoprecipitation revealed similar levels of 23-nt siRNAs in AGO4 and AGO4-SD plants (Supplemental Fig. S1C). This finding, together with our observation that 23-nt RNAs appear to be efficiently sliced once 24/23 siRNA duplexes are loaded into AGO4 (see Figs. 1F, 2A), suggests that most 23-nt siRNAs in cells exist within a pool of 24/23 duplexes that have not been loaded into AGO4. This interpretation is supported by our prior RNA blot results showing that 23- and 24-nt siRNAs are equally sensitive to digestion by dsRNA-specific nuclease V1 and equally insensitive to ssRNA-specific RNase A (Blevins et al. 2015).

Small RNAs that coimmunoprecipitate with both AGO4 and AGO4-SD have a strong signature for a 5′-terminal adenosine (A) among 24-nt siRNAs (Supplemental Fig. S2A). In contrast, the abundant 23-nt siRNAs that copurify with AGO4-SD have a strong signature for uridine (U), located 1 nt upstream of the 3′ terminus (Supplemental Fig. S2B). This U occurs in the RDR2 transcribed RNA strand and is the complement of the 5′-terminal A of the Pol IV transcribed 24-nt siRNA strand (Singh et al. 2019; Loffer et al. 2022). Its penultimate position is due to RDR2's terminal transferase activity (Blevins et al. 2015), which adds an extra untemplated nucleotide onto the 3′ end of its transcripts, thus generating a 1-nt overhang relative to the Pol IV strand. Collectively, the 5′ A and penultimate 3′ U signatures are indicative of 24/23 siRNA duplexes that come from the end of the dsRNA precursor that includes the 5′ end of the Pol IV strand and the 3′ end of the RDR2 strand (see Fig. 1A; Singh et al. 2019).

RNAs of 22, 23, 25, or 26 nt, detected in trace amounts in association with wild-type AGO4, all have strong 5′ A signatures, just like 24-nt siRNAs (Supplemental Fig. S2A). Importantly, the 23-nt RNAs associated with wild-type AGO4 lack the penultimate 3′ U signal characteristic of unsliced 23-nt siRNAs associated with AGO4-SD (cf. Supplemental Fig. S2A,B). Thus, the rare 23-nt siRNAs that associate with wild-type AGO4 are apparently 24-nt siRNAs that were truncated by 1 nt; they are not 23-nt passenger strands. Likewise, the 25- and 26-nt siRNAs found associated with both AGO4 and AGO4-SD appear to be 24-nt siRNAs elongated at their 3′ ends by untemplated uridylation (Supplemental Fig. S2B,C). Our detection of truncated and uridylated forms of 24-nt siRNAs that copurify with AGO4 suggests that siRNAs undergo a turnover process similar to miRNA turnover (Zhao et al. 2012).

### Sliced passenger strand fragments remain associated with AGO4

Approximately 16,200 loci give rise to the 24- and/or 23-nt siRNAs associated with AGO4 or AGO4-SD and account for >95% of the small RNAs detected by sequencing (Supplemental Table S1). A heat map display shows that 12-nt RNAs that coimmunoprecipitate with wild-type AGO4 (see Fig. 2A, top panel) and 23-nt siRNAs that copurify with AGO4-SD are broadly represented among these loci (Fig. 2B).

Genome browser views of individual loci provided insight into the relationships among 12-, 23-, and 24-nt RNAs (Fig. 2C,D). The 12-nt RNAs found associated with wild-type AGO4 come from the same strands as 23-nt RNAs that associate with slicing-defective AGO4. In fact, manual alignment reveals that 12- and 23-nt RNAs share the same 5′ ends (see diagrams at the bottom of Fig. 2C,D). The 3′ termini of 12-nt RNAs occur at the positions where passenger strand slicing is predicted to occur, consistent with 12-nt RNAs being present in wild-type but not slicing-defective AGO4 fractions. Collectively, these data suggest that 12-nt RNAs associated with AGO4 are the 5′ fragments of sliced passenger strand RNAs.

To extend our manual alignments of 23- and 24-nt RNAs at selected loci (e.g., those of Fig. 2C,D) to all loci in an unbiased way, we conducted a computational prediction of strand-pairing patterns among the 5,261,134 unique 24- or 23-nt RNA sequences associated with slicing-defective AGO4 (see Supplemental Fig. S3A; see the Supplemental Material). The pairing configuration represented by the largest number of siRNA pairs has 23-nt siRNAs base-paired to 24-nt siRNAs such that the 5′ ends of the 24-nt strands are recessed by 1 nt relative to the 3′ ends of 23-nt strands (Supplemental Fig. S3B). No such arrangement was observed upon analysis of a computationally simulated library composed of small RNAs with the same abundance and sizes as the experimental data set and coming from same genomic loci but initiating at random positions (Supplemental Fig. S3B,C).

Twenty-four-nucleotide RNAs outnumber 23-nt RNAs by approximately 3:1 in the pool of RNAs associated with slicing-defective AGO4 (Fig. 2A), suggesting that 24-/24-nt and 24-/23-nt duplexes are loaded into AGO4 in similar abundance. Computational analysis of how 24-nt siRNAs can best pair with other, complementary 24-nt siRNAs among the pool of RNAs associated with AGO4-SD predicts duplexes with 2-nt 3′ overhangs at each end as the most abundant duplex species (Supplemental Fig. S3C, left panel), which matches the known pairing of 24/24 duplexes generated by DCL3 dicing in vitro (Fukudome et al. 2021; Loffer et al. 2022). No such pattern was found for 24-nt RNAs associated with wild-type AGO4 (Supplemental Fig. S3C, right panel), consistent with the interpretation that slicing eliminates one strand of any 24/24 duplex that is loaded. It is noteworthy that regardless of whether a passenger strand is 23 or 24 nt in length, slicing will generate a 12-nt product due to cleavage opposite the guide strand's 10th nucleotide when measured from its 5′ end (see Fig. 2E).

### Passenger strand slicing can be recapitulated in vitro using recombinant AGO4

**Figure 3.**
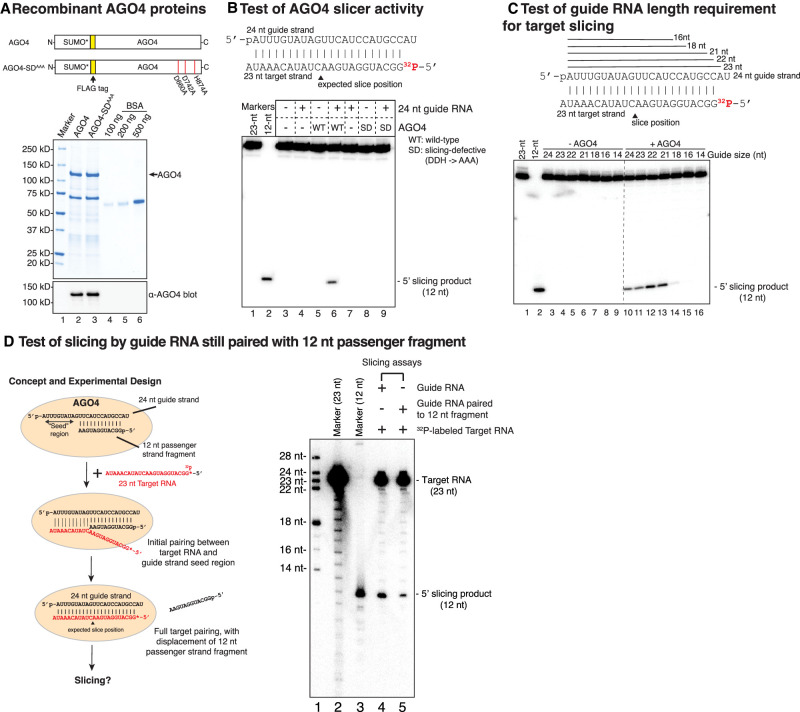
Guide strand-mediated slicing of 23-nt passenger strands can be recapitulated in vitro using recombinant AGO4. (*A*) Diagrams of recombinant AGO4 and AGO4-SD^AAA^ in which aspartates D660 and D742 and histidine H874 were changed to alanines. A Coomassie-stained gel and anti-AGO4 immunoblot reveal similar expression levels for the two proteins. (*B*) AGO4 has passenger strand slicer activity. The diagram shows the pairing of 24- and 23-nt strands of a DCL3-diced 24/23 duplex. The predicted cleavage site is denoted by a black triangle. In the experiment, AGO4 or AGO4-SD^AAA^ was first incubated with or without the 24-nt siRNA guide, and then ^32^P end-labeled 23-nt siRNA was added. Slicing results in a 12-nt end-labeled fragment. RNAs were resolved by denaturing PAGE. End-labeled 23- and 12-nt size markers are present in lanes *1* and *2*. Control reactions lacked added protein (lanes *3*,*4*,*7*) or guide RNA (lanes *3*,*5*,*8*). (*C*) Guide RNAs of variable size program target RNA cleavage. Strands of 23-, 22-, 21-, 18-, 16-, and 14-nt RNA that are 3′ truncations of the 24-nt guide strand shown are represented by solid lines. In the experiment, wild-type AGO4 was preincubated with guide strands of variable length and then end-labeled 23-nt target RNA. End-labeled 23- and 12-nt size markers were run in lanes *1* and *2*. AGO4 was omitted from reactions in lanes *3–9* and included in the reactions of lanes *10–16*. See also Supplemental Figures S4 and S5. (*D*) Test of slicing by a guide RNA paired with a 12-nt passenger strand fragment. The cartoon at the *left* illustrates the unpaired seed region of the guide RNA initiating pairing with a target RNA, leading to displacement of the 12-nt fragment and potential slicing of the target. In the autoradiogram at the *right*, end-labeled size markers were resolved by denaturing PAGE in lanes *1–3*. Recombinant AGO4 was loaded with the 24-nt single-stranded guide RNA (lane *4*) or the guide RNA paired with the 12-nt passenger strand fragment (lane *5*) and tested for the ability to slice a 5′ end-labeled 23-nt target RNA to produce a labeled 12-nt product.

To enable biochemical assays of AGO4 slicing, we used baculovirus vectors to express N-terminal SUMO and FLAG-tagged wild-type or slicing-defective AGO4 in insect cells, with the latter having the catalytic triad mutated from DDH to AAA (abbreviated as AGO4-SD^AAA^) (Fig. 3A). Chaperone activities are usually required for Argonaute loading of double-stranded siRNA duplexes but not single-stranded siRNAs (Iwasaki et al. 2015; Naruse et al. 2018). Thus, to bypass chaperone involvement, we first incubated AGO4 with a single-stranded 24-nt RNA to serve as the guide strand. A complementary 23-nt siRNA 5′ end-labeled with ^32^P (Fig. 3B) was then added, yielding a duplex mimicking a DCL3-generated 24/23 duplex (see the summary diagram in Supplemental Fig, S3B; Loffer et al. 2022). In reactions involving wild-type AGO4, a labeled 12-nt RNA slicing product was generated (Fig. 3B, lane 6), supporting the interpretation that the 12-nt RNAs associated with wild-type AGO4 in vivo (Fig. 2C–E) are slicing products. Recombinant AGO4-SD^AAA^ displayed no slicer activity, as expected (Fig. 3B, lane 9).

We next tested the guide strand length requirements for AGO4 slicing. Interestingly, 21- to 24-nt guide RNAs, differing only at their 3′ ends, programmed equivalent levels of passenger strand slicing (Fig. 3C, lanes 10–13). These results indicate that AGO4 does not anchor the guide RNA's 3′ end at a fixed position within the PAZ domain, unlike human AGO2 and several other AGO proteins (Ma et al. 2004; Jinek and Doudna 2009; Frank et al. 2012). Examination of a predicted structure for AGO4, obtained using AlphaFold (Jumper et al. 2021), suggests that an extended β hairpin and disordered positively charged loop flank the predicted 3′ end of the guide strand RNA in lieu of a nucleotide binding pocket, as in human AGO2 and *Arabidopsis* AGO1 (Supplemental Fig. S4A). The sequences of the disordered loop are also conserved in the AGO4-related proteins AGO6 and AGO9 (Supplemental Fig. S4B; see also Poulsen et al. 2013). We speculate that these extended motifs allow for siRNA 3′ ends of variable length.

A 12-nt passenger strand fragment base-paired to a 24-nt guide strand would leave the seed region of the guide strand (nucleotide positions 2–8, counting from the 5′ end) single-stranded and potentially still able to base-pair with a target RNA (see the left panel in Fig. 3D). To test this possibility, we annealed a 24-nt guide strand with an excess of a 12-nt RNA, equivalent to a sliced passenger strand fragment, and then incubated the resulting 24-/12-nt duplex with recombinant AGO4. A 23-nt target RNA 5′ end-labeled with ^32^P and complementary to the 24-nt guide RNA was then incubated with the complex. Slicing of the 23-nt RNA ensued, with the slicing activity of AGO4 loaded with a 24-/12-nt duplex being comparable with that of AGO4 loaded with only the 24-nt guide RNA (Fig. 3D, cf. lanes 4 and 5).

### Guide strand end modifications and terminal nucleotide requirements

**Figure 4.**
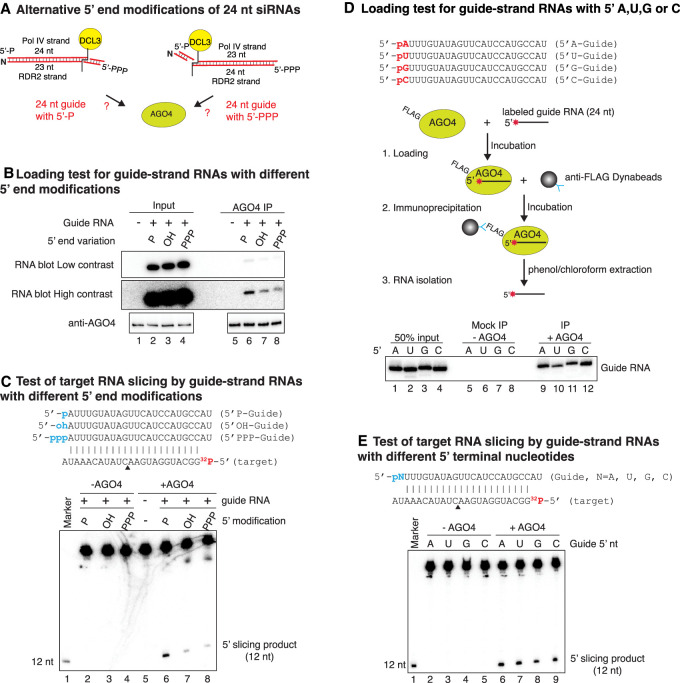
Tests of guide strand 5′ chemical groups and nucleotides in AGO4 loading and slicing. (*A*). Cartoon illustrating alternative features of precursors and diced siRNA duplexes. (*B*) Test of AGO4 loading for 24-nt guide RNAs with 5′ monophosphate (P), 5′ hydroxyl (OH), or 5′ triphosphate (PPP) groups. Input samples are shown in lanes *1–4*. RNAs that coimmunoprecipitate with AGO4 are in lanes *5–8*. RNAs were subjected to denaturing PAGE, transferred to membranes, and detected by RNA blot hybridization. Controls in lanes *1* and *5* lacked RNA. (*C*) Target RNA slicing programmed by guide siRNAs with different 5′ end modifications. Twenty-four-nucleotide guide RNAs with different 5′ groups, paired with a 5′ end-labeled 23-nt target RNA, are shown at the *top*. The predicted slice site is denoted by a black triangle. The alternative guide RNAs were incubated with or without AGO4, followed by incubation with ^32^P-labeled 23-nt target RNA. RNAs were then subjected to denaturing PAGE. An end-labeled 12-nt size marker was run in lane *1*. Lane *5* is a control in which AGO4 was included but guide RNA was omitted. (*D*) Test for preferential loading of guide RNAs with different 5′ nucleotides. Twenty-four-nucleotide RNAs with each of the four possible nucleotides at their 5′ ends were ^32^P end-labeled and incubated with FLAG-tagged recombinant AGO4. (Lanes *9–12*) RNAs associated with AGO4 captured on anti-FLAG Dynabeads were resolved by denaturing PAGE. (Lanes *5–8*) In mock IP control reactions, AGO4 was omitted. Lanes *1–4* show input RNA. See also Supplemental Figure S6. (*E*) Test of target RNA slicing programmed by guide RNAs with different 5′ nucleotides. The different guide RNAs were incubated with (lanes *6–9*) or without (lanes *2–5*) AGO4 and then incubated with end-labeled 23-nt target RNA. RNAs were then purified and subjected to denaturing PAGE and autoradiography.

Results of previous studies suggested that Pol IV transcribed strands of siRNA precursor dsRNAs have 5′ monophosphates (Blevins et al. 2015; Li et al. 2015; Zhai et al. 2015), whereas RDR2 transcribed strands have 5′ triphosphates (see Fig. 4A; Singh et al. 2019). These end groups persist in diced siRNAs (Singh et al. 2019). To test whether 5′ end modifications affect guide strand loading or activity, we incubated FLAG-tagged recombinant AGO4 with 24-nt RNAs having either 5′ hydroxyl, 5′ monophosphate, or 5′ triphosphate groups. Following affinity capture of AGO4 using anti-FLAG Dynabeads, associated RNAs were subjected to RNA blot analysis. The results show that guide RNAs can be loaded into AGO4 regardless of their 5′ end modifications, but the strongest signals were observed for RNAs with a 5′ monophosphate group (Fig. 4B, cf. lanes 6 and 7,8). Guide RNAs with 5′ monophosphate groups also programmed fivefold to eightfold higher levels of target slicing than guide RNAs with 5′ hydroxyl or 5′ triphosphate groups (Fig. 4C, cf. lanes 6 and 7,8). The observed 5′ monophosphate preference suggests that 24-nt siRNAs derived from the 5′ ends of Pol IV strands may be preferentially loaded into AGO4 and may preferentially program slicing compared with siRNAs derived from the 5′ triphosphorylated RDR2 strand. MID domain amino acids known to be involved in 5′ phosphate interactions in *Arabidopsis* AGO1 are highly conserved in sequence and spatial positions in AGO4 (Supplemental Fig. S5A) and all *Arabidopsis* AGO proteins (Supplemental Fig. S5B).

Approximately 80% of the 24-nt siRNAs associated with AGO4 in vivo begin with adenosine, suggesting that AGO4 may selectively bind 24-nt siRNAs with a 5′ A. As a test of this hypothesis, we synthesized 24-nt RNAs that differ only by having 5′ A, U, C, or G. We then end-labeled the RNAs with ^32^P, incubated them with FLAG-tagged recombinant AGO4, affinity-captured the resulting AGO4–RNA complexes, and subjected the RNAs to denaturing PAGE and autoradiography. This experiment revealed that 5′ nucleotide identity has little effect on AGO4 binding (Fig. 4D, lanes 9–12). We also performed a competition assay in which AGO4's binding of a ^32^P-labeled 24-nt RNA with a 5′ A was challenged by inclusion of increasing amounts of phosphorylated but unlabeled 24-nt siRNAs that have A, U, G, or C nucleotides at their 5′ ends. All four competitor RNAs competed effectively against the labeled RNA (Supplemental Fig. S6). Likewise, guide RNAs with 5′ A, U, C, or G nucleotides programmed similar levels of target RNA slicing (Fig. 4E). Collectively, the data of Figure 4 and Supplemental Figure S6 indicate that 5′-terminal nucleotide identity has little effect on AGO4 guide strand loading or enzymatic activity.

### AGO4 retains sliced target RNA fragments in vitro and in vivo

**Figure 5.**
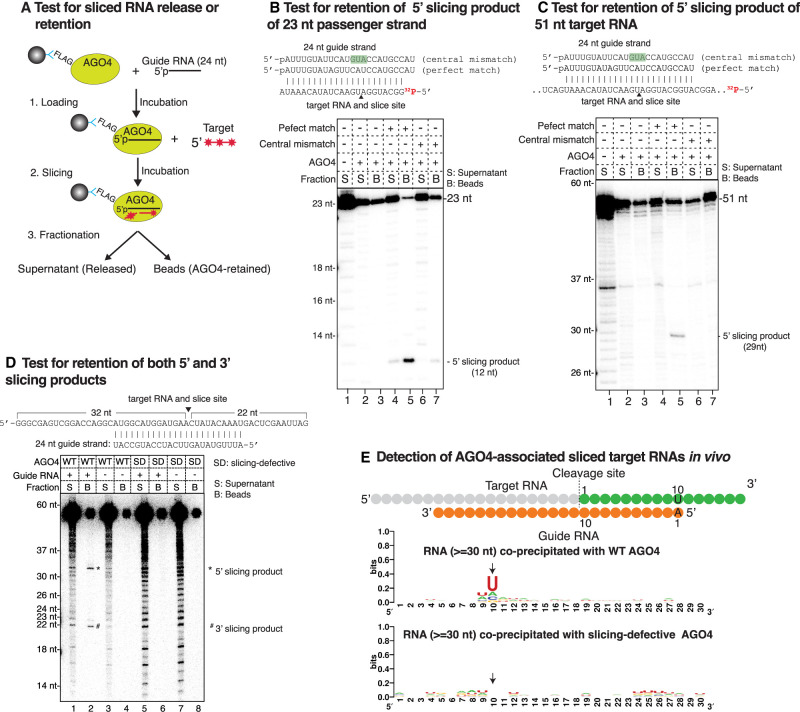
AGO4 retains sliced target RNAs in vitro and in vivo. (*A*) Cartoon of the strategy for testing AGO4 release or retention of sliced target RNAs. (*B*) Test for retention of 23-nt target strand 5′ cleavage products. At the *top* are sequences of a 24-nt guide RNA with 100% complementarity to the ^32^P-labeled target RNAs and a guide RNA with 3 nt of target noncomplementarity (highlighted in green). In the experiment, FLAG-AGO4 immobilized on anti-FLAG Dynabeads was incubated with buffer only (lanes *2*,*3*), with the 24-nt guide RNA that have perfect complementarity to the target RNA (lanes *4*,*5*), or with 24-nt guide RNAs with mismatches to the target RNA (lanes *6*,*7*). RNAs in the supernatant (S) and washed bead (B) fractions were purified and subjected to denaturing PAGE and autoradiography. Lane *1* is an anti-FLAG IP control using nontransgenic plants. (*C*) Test for retention of 5′ cleavage products of a 51-nt RNA target. The experiment was conducted as for *B*. (*D*) Both 5′ and 3′ cleavage products of long RNAs are retained by AGO4 in vitro. The experiment was conducted as for *B* except that a body-labeled 54-nt target RNA was used. The expected sizes of its slicing products are 22 and 32 nt. (*E*) Sliced products of RdDM target locus RNAs are retained by AGO4 in vivo. The cartoon shows how 24-nt guide RNAs of variable sequence (orange) would pair with complementary target RNAs. 5′ and 3′ cleavage products are color-coded gray and green, respectively. At position 10 of the 3′ slicing products (green fragments), a strong uridine (U) signature is expected due to complementarity to the adenosine (A) at the guide strand 5′ end. The sequence logos *below* show RNA-seq analyses for RNAs >30 nt that coimmunoprecipitated with wild-type AGO4 or AGO4-SD. The arrow denotes the position of the expected U signature.

Our detection of 12-nt passenger strand fragments in association with AGO4 led us to test the extent to which sliced RNA fragments are released or retained by AGO4 (Fig. 5). For these experiments, FLAG-tagged AGO4 expressed in transgenic plants was immobilized on anti-FLAG Dynabeads and loaded with a 24-nt RNA guide strand. A complimentary 5′ end-labeled target RNA was then added. After incubation, reactions were separated into supernatant and bead-associated fractions, with the bead fractions subjected to multiple rounds of washing. RNAs of both fractions were then purified, resolved by denaturing PAGE, and visualized by autoradiography (Fig. 5A). Using a 23-nt target RNA mimicking a passenger strand, an end-labeled slicing product of 12 nt was detected primarily in the washed bead fraction, not the supernatant (Fig. 5B, cf. lanes 4 and 5). Changing guide strand nucleotides 9–11 to make them noncomplementary to the corresponding 23-nt target RNA nucleotides impaired slicing, demonstrating the importance of base pairing near the cut site (Fig. 5B, cf. lanes 6,7 and 4,5). Next, we tested AGO4's ability to slice a 51-nt target RNA end-labeled with ^32^P (Fig. 5C), yielding a labeled 29-nt fragment that remained associated with bead-immobilized AGO4 (Fig. 5C, cf. lanes 5 and 4).

Using 5′ end-labeled target RNAs only allows 5′ slicing products to be detected. This led us to test a body-labeled 54-nt target RNA generated via incorporation of an α-^32^P-labeled nucleotide during transcription by T7 RNA polymerase in vitro. Labeled fragments of 32 and 22 nt were generated upon AGO4 slicing, corresponding to both the 5′ and 3′ cleavage products (Fig. 5D, lane 2), and both were enriched in the AGO4 bead fraction. This experiment indicates that both 5′ and 3′ slicing products remain associated with AGO4 after target RNA cleavage. Control experiments confirmed that slicing products are not generated in the absence of a guide RNA (Fig. 5D, lane 4) or by slicing-defective AGO4 (Fig. 5D, lanes 6,8).

Our finding that AGO4 retains its slicing products in vitro prompted us to ask whether scaffold RNAs at RdDM loci, primarily synthesized by RNA polymerase V (Wierzbicki et al. 2009), are also sliced and retained in vivo. A prediction is that sliced scaffold RNAs would include RNAs with a strong signature for a uridine (U) at position 10. This is because AGO4-loaded 24-nt guide RNAs have an ∼80% bias for a 5′-terminal adenosine (A) and because slicing occurs opposite the 10th position measured from this 5′ A. Thus, the expected U at position 10 of a sliced target RNA would be the complement of the guide strand's 5′-terminal A (see the model in the top panel of Fig. 5E). With this prediction in mind, we analyzed the sequences of all RNAs that coimmunoprecipitated with wild-type AGO4 in vivo, examining RNAs of ≥30 nt. A strong signature for U is indeed present at position 10 of these AGO-associated RNAs (Fig. 5E). No +10 U signature was observed for RNAs associated with AGO4-SD (Fig. 5E).

### AGO4 slicer activity elevates DNA methylation levels at RdDM loci genome-wide

**Figure 6.**
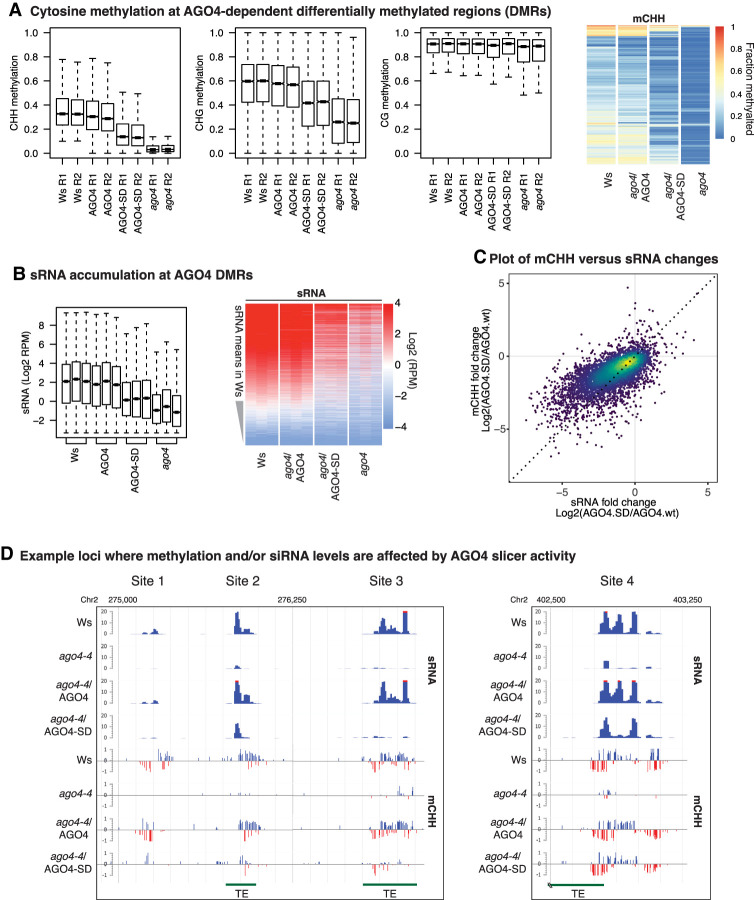
AGO4 slicer activity enhances non-CG methylation at virtually all RdDM loci. (*A*) Cytosine methylation at AGO4-dependent differentially methylated regions (DMRs). (*B*) Relative small RNA abundance at AGO4 DMRs displayed as box plots and as a heat map. siRNA accumulation was normalized to the total mappable read count of the corresponding small RNA library. (RPM) Reads per million. (*C*) Correlation between CHH methylation and siRNA levels at AGO4 DMRs. Points overlapping at higher density are shown in warmer colors. (*D*) Representative genome browser views of loci at which de novo DNA methylation and/or small RNA accumulation is affected by AGO4 slicer activity. See also Supplemental Figure S7.

We tested the importance of AGO4's slicing ability for DNA methylation at RdDM loci using whole-genome bisulfite sequencing. In *ago4-4*-null mutant plants, compared with wild-type plants, cytosine methylation at CHH and CHG motifs is substantially reduced at 6718 genomic loci, whereas CG methylation is mostly unaffected (Fig. 6A, cf. left three panels). We define these 6718 loci as AGO4-dependent differentially methylated regions (agoDMRs) (see Supplemental Table S2). Rescue of the *ago4-4* mutant by the wild-type AGO4 transgene restored CHG and CHH methylation to nearly wild-type levels. In contrast, expression of AGO4-SD resulted in CHG and CHH methylation levels intermediate between those of the mutant and wild type, and this was true for the majority of agoDMR loci (see heat map panel of Fig. 6A). AGO4-dependent CHG and CHH methylation is particularly apparent at short TEs (1–2 kb) and the edges of long TEs (>4 kb) (Supplemental Fig. S7), consistent with RdDM mainly targeting these regions (Zemach et al. 2013).

Analyses of siRNA levels by RNA-seq revealed that in the *ago4-4* mutant, siRNA levels at agoDMR loci decrease, paralleling the decreases in CHG and CHH methylation levels. Like cytosine methylation, siRNA levels are nearly fully restored by wild-type AGO4 but are only partially restored by AGO4-SD (Fig. 6B). The positive correlation between CHH methylation levels and small RNA levels is illustrated in Figure 6C.

The data of Figure 6, A and B, and Supplemental Figure S7 suggest that AGO4 slicing has primarily quantitative, rather than qualitative, effects on siRNA and CHH methylation levels at agoDMR loci. However, within individual loci, differences in the spatial distribution of siRNAs and CHH methylation are often apparent, as shown in genome browser views for four loci (Fig. 6D). At sites 1 and 3, CHH methylation levels are severely impacted in the *ago4-4* mutant and are restored to wild-type levels by the wild-type AGO4 transgene but are only weakly rescued by the AGO4-SD transgene, which also fails to restore siRNA levels. In comparison, sites 2 and 4 have two or three spatially separated peaks of siRNAs that are greatly diminished in the *ago4-4* mutant and are restored by wild-type AGO4. At each of these loci, the AGO4-SD protein fails to restore one of the siRNA peaks, which correlates with reduced methylation within that interval (most apparent for site 2). Collectively, these results indicate that quantitative differences in slicing-dependent methylation and siRNA levels at RdDM loci can frequently be attributed to differences within subintervals of affected loci.

## Discussion

### The function of 23-nt siRNAs revealed

DCL3-generated 23-nt RNAs derived from the 3′ ends of Pol IV or RDR2 strands of siRNA precursors are base-paired to 24-nt siRNAs derived from precursor 5′ ends (Singh et al. 2019; Loffer et al. 2022), but whether 23-nt RNAs play a role in RdDM has long been a question. Our results provide an answer, showing that 23-nt RNAs function as passenger strands for 24-nt siRNA guide strands. The 24-nt guide strands then program AGO4 to slice the 23-nt passenger strands with nearly 100% efficiency. What few 23-nt RNAs remain associated with AGO4 lack the 3′ penultimate U signature of passenger strands; instead, they have sequences indicative of truncated 24-nt strands (see Supplemental Fig. S2). Collectively, our results indicate that 23-nt siRNAs do function in RdDM but only indirectly—by specifying that their 24-nt partners are loaded into AGO4 to carry out subsequent target locus interactions.

### Old and new hypotheses for AGO4 guide strand selection

How is the 24-nt strand of a 24/23 duplex selected as the guide strand? A long-standing hypothesis has been that AGO4 has a structural requirement for 24-nt RNAs such that 23-nt RNAs cannot be stably bound. However, our biochemical results show that AGO4 can be loaded with 21-, 22-, 23-, or 24-nt RNAs, all of which guide target RNA slicing with similar efficiency (see Fig. 3). Intriguingly, low-abundance Pol IV-dependent 21- to 22-nt siRNAs are generated at loci where 24-nt siRNAs accumulate and can participate in RdDM (Panda et al. 2020). Our biochemical results support the hypothesis that these 21- to 22-nt siRNAs could potentially be loaded into AGO4, as is the case for AGO6, which incorporates 21- to 22-nt TE-derived siRNAs and guides DNA methylation in the noncanonical RdDM pathway (McCue et al. 2015).

It has also been thought that AGO4 may actively select guide strands with a 5′-terminal adenosine because ∼80% of 24-nt siRNAs associated with AGO4 have 5′ adenosines (Mi et al. 2008). Precedence for this idea includes structural studies of human AGO2, whose nucleotide specificity loop in the MID domain favors binding of a 5′ U or A (Frank et al. 2010, 2012), and studies of *Arabidopsis* AGO1, whose similar specificity loop favors binding of miRNAs with a 5′ U (Mi et al. 2008; Frank et al. 2012). However, our results show that 24-nt siRNAs with 5′ A, U, C, or G can associate with AGO4 and mediate target RNA cleavage with similar efficiencies (see Fig. 4D,E). Taken together, our tests of guide strand length and terminal nucleotide identity lead us to conclude that AGO4 binds the siRNAs available to it, as dictated by the activities of the enzymes that generate the siRNAs; namely, Pol IV, RDR2, and DCL3 (Singh et al. 2019). Pol IV most frequently initiates transcription with an A or G; likewise, RDR2 has a (weaker) tendency to initiate with an A or U (Singh et al. 2019). Thus, enrichment for 5′ adenosines is a feature of siRNA precursors. DCL3 then displays a preference for binding dsRNA ends that include a 5′ A (and discriminates against a 5′ G) and then measures and cuts 24 nt downstream, resulting in biased production of 24-nt siRNAs with a 5′ A (Loffer et al. 2022). However, the question of why 24nt and not-23 nt RNAs of 24/23 duplexes are selected as guide strands remains. The asymmetry inherent to 24/23 duplexes is likely important, with 3′ ends of 23-nt strands overhanging by 1 nt and 3′ ends of the 24-nt strands overhanging by 2 nt. We speculate that this asymmetry is recognized by an as yet unidentified AGO4 loading activity that binds and orients the duplex such that the 24-nt strand is stably incorporated into AGO4. In the case of 24/24 duplexes that are also generated by DCL3 and are composed of the 3′ ends of Pol IV strands and the 5′ ends of RDR2 strands (Loffer et al. 2022), 3′ overhangs of 2 nt are present at each end such that these duplexes are symmetrical. If a 3′ overhang of 2 nt is what identifies the strand as loaded, the 24-nt strand of a 24/23 duplex would be specifically chosen, but either strand of a 24/24 duplex could be chosen.

### Retention of sliced RNAs suggests a model for how slicing may enhance RdDM

Our finding that 12-nt 5′ fragments of sliced 23-nt passenger strands remained associated with AGO4 both in vivo and in vitro (see Figs. 2, 5B) was unexpected. Presumably, the 12-nt fragments are base-paired to the 3′ half of 24-nt guide strands, leaving the seed region of the guide strands single-stranded and available to initiate pairing with complementary target RNAs. Our test of this hypothesis showed that a 24-nt guide strand–12-nt passenger strand fragment duplex can indeed program target RNA slicing (see Fig. 3D). We speculate that the 12-nt fragment is displaced upon target RNA binding, given that slicing requires perfect guide strand–target RNA base pairing surrounding the slice site (see Fig. 5B,C) and that the 12-nt RNA overlaps this region.

An unanswered question is whether the 12-nt passenger strand slicing product is important for some aspect of AGO4 function. One possibility is that it helps protect the 3′ end of the paired 24-nt guide RNA and may be accommodated by the AGO4 PAZ domain's predicted extended hairpin and disordered loop, which differ from the end-tethering pockets of other AGOs (see Supplemental Fig. S4). Another possibility is that a 12-nt passenger strand fragment base-paired to a guide strand serves as a mark of a new and unused AGO4–siRNA complex that has not yet seen action by engaging a complementary target RNA. Such a mark could potentially play a role in subcellular trafficking or docking of AGO4–siRNA complexes with Pol V or other components of the RdDM machinery.

A pioneering study indicated that AGO4 slicing competence affected methylation and siRNA abundance at some but not all of a limited set of *Arabidopsis* RdDM loci known at the time (Qi et al. 2006). Revisiting the question using bisulfite sequencing and small RNA deep sequencing to achieve a whole-genome analysis shows that AGO4 slicer activity is needed to achieve maximal DNA methylation and siRNA levels at nearly all RdDM loci, not just a specific subset of loci (Fig. 6A,B; Supplemental Fig. S7). What does AGO4 slice? Early studies showed that AGO4 can be cross-linked to Pol V transcripts and that Pol V transcript levels detected by RT-PCR are higher in *ago4* mutants (Wierzbicki et al. 2009). Thus, Pol V transcripts have long been assumed to be likely targets of AGO4 slicing. Moreover, RNA sequencing of nascent Pol V transcripts generated under nuclear run-on conditions revealed a strong +10 U signature that, together with genetic evidence, suggested cotranscriptional slicing by one or more members of the AGO4 clade (Liu et al. 2018). Our study adds to these previous results by showing that AGO4 can account for the +10 U signature and that AGO4 surprisingly remains associated with the sliced products of nascent Pol V transcripts.

**Figure 7.**
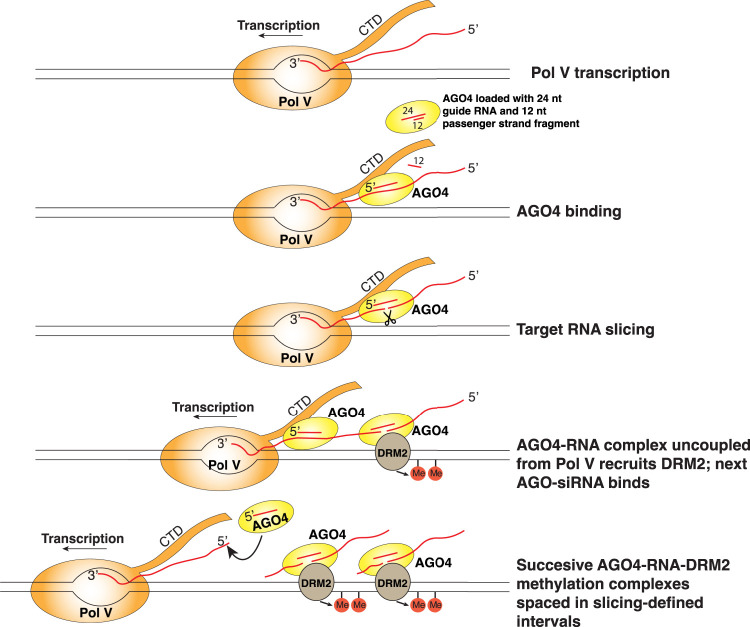
A model for discontinuous RNA-directed DNA methylation in intervals reflecting AGO4 slicing.

Why should AGO4 retain its slicing products? Importantly, Pol V transcripts are thought to be >100 nt (Böhmdorfer et al. 2016) and overlap genomic positions at which 24-nt siRNAs are generated in swarms (Wierzbicki et al. 2012), beginning and ending at variable positions throughout the loci (see Fig. 2C,D). Thus, multiple 24-nt siRNAs can potentially base-pair with any given Pol V transcript. There is also compelling evidence that protein–protein interactions occur between the C-terminal domain of the Pol V largest subunit and AGO4 (El-Shami et al. 2007), likely in a complex also involving AGO4 interactions with SPT5L (Lahmy et al. 2016; Liu et al. 2018). If AGO4–CTD interactions and siRNA–Pol V transcript interactions occur simultaneously and cotranscriptionally, can these interactions be maintained as Pol V transcription elongation proceeds and the distance (measured in RNA length) between the polymerase and an AGO4–siRNA complex progressively increases? Based on these considerations, we propose a model, shown in Figure 7, in which protein–protein interactions between the Pol V CTD and AGO4 initially occur coincident with siRNA–Pol V transcript base pairing. AGO4 slicing of the Pol V transcript ensues, allowing the 5′ portion of the nascent transcript to be uncoupled from the still elongating polymerase, with the AGO4–siRNA complex remaining bound to the sliced and released RNA. This process might then be repeated by the binding, slicing, and release of successive AGO4–siRNA complexes as Pol V transcription proceeds. A further speculation is that the sliced RNA fragments retained by AGO4 may facilitate direct tethering at the corresponding DNA loci, perhaps contributing to evidence for R loops at RdDM loci (Xu et al. 2017). Mass spectrometry evidence indicates that AGO4 and the de novo DNA methyltransferase DRM2 can associate (Zhong et al. 2014). Thus, individual tethered AGO4–RNA complexes may be sufficient for DRM2 recruitment and methylation of corresponding DNA intervals, with methylation able to proceed at a pace independent of the rate of Pol V transcriptional elongation thanks to AGO4 slicing. Our finding that cytosine methylation and associated siRNA production can differ within RdDM locus subintervals that can also differ in their dependence on AGO4 slicing ability (see Fig. 6) is compatible with the hypothesis that multiple AGO4–DRM complexes may act in parallel in intervals at least partially defined by AGO4 slicing. Testing the model of Figure 7 will be a challenge for future studies.

## Materials and methods

### AGO4 protein expression and purification

AGO4 proteins used in this study were expressed in *A. thaliana* or insect cells and purified as detailed in the Supplemental Material. Purified AGO4 proteins were resolved in 4%–20% miniprotean stain-free gels (Bio-Rad) and visualized by Coomassie blue staining or immunoblotting with HRP-conjugated anti-FLAG M2 antibodies (Sigma) or anti-AGO4 polyclonal antibodies (Pikaard laboratory antibody stock no. 18187).

### Analysis of AGO4-bound RNAs

RNAs copurifying with wild-type AGO4 or AGO4-SD from *A. thaliana* were either 5′ end-labeled by [γ-^32^P] ATP and resolved in polyacrylamide gels under denaturing condition or subjected to high-throughput RNA sequencing. Detailed methods for RNA preparation are described in the Supplemental Material.

### RNA oligonucleotide preparation

RNA oligos (Supplemental Table S3) were synthesized by Integrated DNA Technologies (IDT), except for the triphosphorylated oligo used in Figure 4, B and C, which was synthesized by BioSynthesis, Inc. RNA oligos were gel-purified (Nilsen 2013). Purified RNA oligos were either phosphorylated with unlabeled ATP or 5′ end-labeled using [γ-^32^P] ATP (Perkin Elmer) and T4-polynucleotide kinase (New England Biolabs), followed by purification using Performa spin columns (EdgeBio). ^32^P-labeled RNA oligos were gel-purified again to remove degraded RNAs.

### In vitro guide RNA loading and target RNA slicing assay

Immunoprecipitated AGO4 from transgenic *A. thaliana* or partially purified AGO4 from insect cells (detailed in the Supplemental Material) were incubated with 40 nM unlabeled phosphorylated guide RNA in 20 µL of reaction buffer (100 mM potassium acetate, 25 mM HEPES-KOH at pH 7.4, 5 mM magnesium acetate, 10% glycerol, 0.01% Igepal, 0.5 mM DTT, 1 mM PMSF) for 1 h at room temperature to allow guide RNA incorporation into AGO4. One microliter of ∼1000 cpm/µL ^32^P-phosphorylated target RNA and 1 µg of yeast tRNA (Thermo Fisher Scientific) were added to each reaction. After 1 h of incubation at room temperature, reactions were quenched by adding 200 µL of stop solution (300 mM NaOAc, 7 M urea). RNAs were extracted by 200 µL of phenol/chloroform/isoamyl alcohol, ethanol-precipitated, resuspended in 1× formamide loading buffer (40% deionized formamide, 0.5 mg/mL xylene cyanol, 0.5 mg/mL bromophenol blue, 5 mM EDTA at pH 8.0), resolved in 15% polyacrylamide gels containing 7 M urea, and visualized by phosphorimaging.

### Small RNA sequencing and data analyses

RNAs coimmunoprecipitating with AGO4 in inflorescence tissues were purified and used to generate small RNA sequencing libraries using Illumina's TruSeq small RNA library preparation kit. The cDNA libraries were single-end-sequenced using an Illumina NextSeq500 platform. The 3′ adapter sequences were removed using Cutadapt v1.18 (Martin 2011) with the following options: -a TGGAATTC –discard-untrimmed -e 0 -m 8 -O 8. Trimmed reads were aligned to the *A. thaliana* TAIR10 genome using ShortStack v3.8 (Johnson et al. 2016), allowing no more than two mismatches. The alignment files were loaded in JBrowse configured with “small RNA plug-in” (Hofmeister and Schmitz 2018) with modifications to allow customized small RNA alignment visualization. Sequence logos were prepared using WebLogo 3.6 (Crooks et al. 2004). Heat maps for siRNA loci where 24-nt siRNAs are the predominant class were clustered by complete linkage with Euclidean distances calculated. Detailed methods for in silico small RNA duplex reconstitution are in the Supplemental Material.

### Whole-genome bisulfite sequencing and methylome data analysis

Genomic DNA was extracted from inflorescence tissues using a Nucleon Phytopure DNA extraction kit (Cytiva). Whole-genome bisulfite sequencing libraries for two biological replicates of each genotype were prepared using Perkin Elmer's Nextflex bisulfite-seq kit with ∼500 ng of fragmented genomic DNA. Unmethylated cytosines in the adapter-ligated DNA were chemically converted by EZ DNA Methylation-Gold kit (Zymo Research Corp.). The CT-converted products were purified and subjected to 12 cycles of PCR amplification before single-end sequencing using an Illumina NextSeq500 platform.

To analyze the whole-genome bisulfite sequencing data, 3′ adapter sequences were first removed by Cutadapt v1.18 (Martin 2011) with the following options: -a AGATCGGAAGAGCACACGTCTGAACTCCAGTCAC -m 15 -q 20. Trimmed reads were aligned to the *Arabidopsis* TAIR10 genome using bsmap2 (Xi and Li 2009) with the following options: *-*w 100 -n 1 -p 3 -v 0.08. The *Arabidopsis* genome sequence was binned into 100-nt windows, with CG, CHG, and CHH (where H represents any nucleotide except G) methylation rate calculated for each window. To avoid regions with low sequencing coverage, only bins with at least four cytosines that were each covered by no less than four reads were included in the analysis. Bins that had at least 10% less CHH methylation in two independent replicates of *ago4-4* compared with two independent replicates of wild-type plants were defined as differentially methylated bins. Differentially methylated bins within 200 bp from each other were then merged into differentially methylated regions (DMRs).

### Data availability

AGO4 immunoprecipitated RNA sequencing and whole-genome bisulfite sequencing data sets have been deposited at GEO with accession number GSE201235. In-house scripts for these analyses are available at https://github.com/wangfeng3392/small_RNA.

### Additional materials and methods

Additional materials and methods are available in the Supplemental Material.

## Supplementary Material

Supplemental Material
