# Supplementary material for: Enzymatic reactions of AGO4 in RNA-directed DNA methylation: siRNA duplex loading, passenger strand elimination, target RNA slicing, and sliced target retention
Source: Genes Dev. 2023 Feb 1;37(3-4):103–18. doi: 10.1101/gad.350240.122 (PMC10069450; doi:10.1101/gad.350240.122)
Supplement: Supplemental Material [file supp_37_3-4_103__DC1.html]

Enzymatic reactions of AGO4 in RNA-directed DNA methylation: siRNA duplex loading, passenger strand elimination, target RNA slicing, and sliced target retention — Enzymatic reactions of AGO4 in RNA-directed DNA methylation: siRNA duplex loading, passenger strand elimination, target RNA slicing, and sliced target retention — Supplemental Material 

# Enzymatic reactions of AGO4 in RNA-directed DNA methylation: siRNA duplex loading, passenger strand elimination, target RNA slicing, and sliced target retention

## Supplemental Material

- Supplemental\_Materials\_and\_Methods.docx
- Supplemental\_Figure\_Legends.docx
- Supplemental\_FigS1.pdf
- Supplemental\_FigS2.pdf
- Supplemental\_FigS3.pdf
- Supplemental\_FigS4.pdf
- Supplemental\_FigS5.pdf
- Supplemental\_FigS6.pdf
- Supplemental\_FigS7.pdf
- Supplemental\_TableS1.xlsx
- Supplemental\_TableS2.xlsx
- Supplemental\_TableS3.pdf
