# Supplementary material for: Enzymatic reactions of AGO4 in RNA-directed DNA methylation: siRNA duplex loading, passenger strand elimination, target RNA slicing, and sliced target retention
Source: Genes Dev. 2023 Feb 1;37(3-4):103–18. doi: 10.1101/gad.350240.122 (PMC10069450; doi:10.1101/gad.350240.122)
Supplement: Supplemental Material [file supp_gad.350240.122_Supplemental_Materials_and_Methods.docx]

**Supplemental Materials and Methods**

**AGO4 immunoprecipitation from transgenic Arabidopsis plants**

AGO4 proteins were immunoprecipitated from inflorescence tissues of transgenic *A. thaliana* plants*,* ecotype Wassilewskija (Ws), stably expressing FLAG-tagged wild-type AGO4 or slicing-defective AGO4-SD in the *ago4-4* mutant background. One gram of inflorescence tissue was ground to fine powder in liquid nitrogen and resuspended in 6 mL extraction buffer [50 mM Tris-HCl (pH 7.9), 75 mM sodium chloride, 5 mM magnesium chloride, 10% glycerol, 0.5% IGEPAL, 1 mM DTT, 1 mM PMSF and 1X plant protease inhibitor mix (Millipore Sigma)]. The homogenate was subjected to centrifugation at 16,000 g for 20 minutes at 4 ˚C. The supernatant was collected, filtered through a CellTrics 30 um strainer (Sysmex), and subjected to a second round of centrifugation at 16,000 g for 20 minutes at 4˚C. The supernatant was incubated with 50 μL of anti-FLAG M2 agarose resin (Sigma) for 3 hours using a rotating mixer at 4 ˚C, followed by 4 washes, each with 1 mL of extraction buffer without plant protease inhibitors. The resin was resuspended in extraction buffer (without protease inhibitors) to a final volume of 100 μL. For protein analysis, 10 μL of the slurry containing immunoprecipitated AGO4 was boiled at 95 ^o^C for 5 min in SDS sample buffer, resolved on 4-20% Mini-PROTEAN stain-free gels (Bio-Rad), and visualized by Coomassie Blue staining or immunoblotting with HRP-conjugated anti-FLAG M2 antibodies (Sigma) or anti-AGO4 polyclonal antibodies (Pikaard lab antibody stock #: 18187).

**Extraction and analysis of AGO4-bound RNAs**

Copurifying RNAs were released by incubating 50 μL of the slurry prepared as mentioned above at 70 ^o^C for 5 min, precipitated with ethanol, and subjected to 5’ end-labeling with [γ-^32^P] ATP (Perkin Elmer) and T4 polynucleotide kinase (New England Biolabs). The labeled RNAs were passed through Performa Spin Columns (EdgeBio) to remove unincorporated radioactive ribonucleotides, resolved by polyacrylamide gel under denaturing condition (containing 7 M urea), and visualized by autoradiography. For small RNA sequencing, 25 μL of the slurry containing immunoprecipitated AGO4 was treated with 0.8 unit of proteinase K (New England BioLabs) followed by RNA extraction using TE saturated phenol/chloroform/isoamyl alcohol (pH 4.5). The RNA in the aqueous phase was ethanol precipitated, washed with 70% ethanol, and resuspended in nuclease-free water. The extracted RNAs were then used for high-throughput small RNA-seq library preparation.

**Computational reconstitution of small RNA duplexes**

Pairing patterns among siRNAs were analyzed using an in-house Python script. First, identical small RNAs aligning to the same genomic location were condensed into one sequence. Then, all possible pairs of 24 nt and 23 nt siRNAs that overlap in opposite orientation were analyzed. The distance from the 5’ end of a 5’A-bearing 24 nt siRNA to the 3’ end of the paired 23 nt siRNA (defined as 5’ to 3’ registry) for all 24/23 nt siRNA pairs was calculated and the frequencies of all possible pairing patterns were plotted. The 24/12 nt RNA pairing patterns were analyzed similarly. A simulated small RNA sequencing dataset mimicking the features of the actual small RNA-seq libraries was generated as a control using in-house script. To do so, the read alignment positions were randomized in the simulated libraries, while overall read abundance, read size distribution, and cluster locations were maintained. The simulated dataset was aligned to the *A. thaliana* TAIR10 genome assembly and analyzed in the same way as mentioned above. The in-house scripts for these analyses are available at: https://github.com/wangfeng3392/small_RNA.

***In vitro* slicing assay using immunoprecipitated AGO4 from Arabidopsis plants**

AGO4 was immunoprecipitated from homozygous transgenic lines expressing either FLAG-AGO4 or slicing-defective FLAG-AGO4-SD in the *ago4-4* null mutant background. To prepare AGO4 immunoprecipitation fractions for 8 slicing reactions, two grams of inflorescence tissue was ground to a fine powder in liquid nitrogen and resuspended in 6 mL extraction buffer [400 mM Potassium acetate, 25 mM HEPES-KOH (pH 7.4), 5 mM Magnesium acetate, 10% glycerol, 0.1% IGEPAL, 0.5 mM DTT, 1 mM PMSF and 1X plant protease inhibitor mix (Sigma)]. The homogenate was subjected to centrifugation at 16,000 g for 20 minutes at 4 ˚C. The supernatant was collected, filtered through CellTrics 30 um (Sysmex), and subjected to a second round of centrifugation at 16,000 g for 20 minutes at 4˚C.

FLAG antibody-conjugated Dynabeads for 8 reactions were prepared with the following method: 64 µL Protein G-conjugated Dynabeads (Thermo Fisher Scientific) were incubated with 16 µL monoclonal anti-FLAG M2 antibody (F1804, Millipore Sigma) for no less than 30 minutes at room temperature, or overnight at 4 ˚C. Excess antibody was removed by pulling down Dynabeads using a DynaMag magnet (Thermo Fisher Scientific). The supernatant was discarded, and the beads were washed 3 times with 1 mL extraction buffer [400 mM Potassium acetate, 25 mM HEPES-KOH (pH 7.4), 5 mM Magnesium acetate, 10% glycerol, 0.1% IGEPAL, 0.5 mM DTT, 1 mM PMSF and 1X plant protease inhibitor mix (Sigma)]. To precipitate AGO4 proteins, antibody-conjugated beads were incubated with clarified lysate for 2 hours at 4 ˚C with constant rotation. After removing the lysate, the beads were washed three times with wash buffer [600 mM Potassium acetate, 25 mM HEPES-KOH (pH 7.4), 5 mM Magnesium acetate, 10% glycerol, 0.1% IGEPAL, 1 mM PMSF], and then washed once with reaction buffer [100 mM Potassium acetate, 25 mM HEPES-KOH (pH 7.4), 5 mM Magnesium acetate, 10% glycerol, 0.01% IGEPAL, and 1 mM PMSF]. For each wash, the beads were resuspended by pipetting up and down followed by rotating for 5 minutes at 4 ˚C. The beads were then pulled down using the DynaMag magnet, resuspended in 160 µL reaction buffer containing 1 unit/μL RiboLock RNase Inhibitor (Thermo Fisher), and evenly aliquoted to 8 tubes.

Non-radioactive phosphorylated guide RNA was added to AGO4 loading reactions to a final concentration at 40 nM. The reactions were incubated at room temperature for 1 hour with constant rotation. The beads were then magnet-precipitated and washed three times with reaction buffer to remove unbound guide RNAs. The beads were then resuspended in 20 μL reaction buffer containing 1 unit/μL RiboLock RNase Inhibitor (Thermo Fisher), ^32^P-phosphorylated target RNA (~1000 CPM per reaction), and 1 µg yeast tRNAs (Thermo Fisher Scientific). The reactions were further incubated for 1 hour at room temperature with constant rotation. After incubation, Dynabeads were magnet-precipitated. The supernatant fraction was transferred to a fresh tube. The beads were subject to 3 washes with reaction buffer followed by magnet-precipitation (the bead fraction). The reactions in both supernatant and bead fractions were quenched by adding 200 µL of stop solution (300 mM sodium acetate and 7M urea). RNAs were extracted with 200 µL phenol/chloroform/isoamyl alcohol followed by ethanol precipitation. The resulted RNA pellets were washed once with 70% ethanol, allowed to dry, and resuspended with 1X formamide loading buffer [40% deionized formamide, 0.5 mg/mL xylene cyanol, 0.5 mg/ml bromophenol blue, and 5 mM EDTA (pH 8.0)]. The resuspended RNA samples were then denatured at 95 ˚C and resolved in a 15% polyacrylamide gel containing 7M urea by electrophoresis with a constant wattage of 50 W. The gels were dried and visualized by phosphorimaging.

**Recombinant AGO4 expression and purification**

Codon-optimized N-terminal FLAG-tagged AGO4 or slicing-defective AGO4-SD (with mutations D660A, D742A, and H874A) were first sub-cloned into SUMOstar Insect Intracellular Vector (LifeSensors), and then transformed into *E. coli* strain DH10Bac to generate bacmid DNA. Baculovirus was prepared as previously described (Fitzgerald et al., 2006) except that Sf9 cells cultured in Sf-900 II serum-free media (Thermo Fisher Scientific) was used for virus production. To express recombinant AGO4 and slicing-defective AGO4-SD proteins, 2 L of Sf9 cells with a cell density of approximately 1.5X 10^6^ cells/mL were infected at a multiplicity of infection (MOI) of 2 and further cultured on a shaker at 125 rpm for 48 to 60 hours, at 27 ˚C. Cells were collected by centrifuge at 800 g for 10 minutes, washed once with 1X phosphate buffered saline (PBS) buffer, pelleted again at 800 g for 10 minutes, and flash frozen in liquid nitrogen. All subsequent purification steps were carried out at 4 ˚C. Cell pellets were resuspended in 400 mL ice-cold lysis buffer [400 mM Potassium acetate, 25 mM HEPES-KOH (pH 7.4), 5 mM Magnesium acetate, 10% glycerol, 0.1% IGEPAL, 0.5 mM DTT, 1 mM PMSF and 1X plant protease inhibitor mix (Sigma)]. Cells were disrupted by 8 strokes in a prechilled Dounce Homogenizer. The lysate was clarified by centrifugation at 48,000 g for 45 min followed by immunoprecipitation using 2 mL pre-equilibrated anti-FLAG M2 agarose beads (Sigma) for 2 hours. The beads were subject to 3 washes, each time using 10 mL of wash buffer [400 mM Potassium acetate, 25 mM HEPES-KOH (pH 7.4), 5 mM Magnesium acetate, 10% glycerol, 0.1% IGEPAL, and 1 mM PMSF]. Recombinant AGO4 was eluted 3 times by incubating the beads with 2 mL wash buffer containing 0.5 mg/mL 3X FLAG peptide (APEXBIO) and 20 mM Imidazole. The eluted fraction was further incubated with 0.5 mL of Nickel-NTA agarose beads (Qiagen) for 1 hour. The beads were washed 3 times in the wash buffer containing 40 mM Imidazole. Recombinant AGO4 was eluted 3 times using NiNTA elution buffer [250 mM Imidazole, 400 mM Potassium acetate, 25 mM HEPES-KOH (pH 7.4), 5 mM Magnesium acetate, 10% glycerol, 0.01% IGEPAL, and 1 mM PMSF]. The pooled eluted fraction was dialyzed into storage buffer [150 mM Potassium acetate, 25 mM HEPES-KOH (pH 7.4), 5 mM Magnesium acetate, 10% glycerol, 0.01% IGEPAL, 0.5 mM DTT, 1 mM PMSF], concentrated to ~ 500 ng/µL, divided to 8 µL aliquots, and flash frozen in liquid nitrogen.

***In vitro* slicing using insect cell expressed recombinant AGO4**

Recombinant AGO4 or AGO4-SD (approximately 1 µg each) were incubated with 40 nM of unlabeled phosphorylated guide RNA in 20 µL of reaction buffer containing 1 unit/μL RiboLock RNase Inhibitor (Thermo Fisher) for 1 hour at room temperature to allow guide RNA incorporation into AGO4. 1 µL ^32^P-phosphorylated target RNA (~1000 cpm/µL) and 1 µg yeast tRNA (Thermo Fisher Scientific) was added to each reaction. After 1 hour incubation at room temperature, reactions were quenched by adding 200 µL of stop solution (300 mM NaOAc and 7M urea). RNAs were extracted by 200 µL of phenol/chloroform/isoamyl alcohol, ethanol precipitated, resuspended in 1X formamide loading buffer, resolved in 15% polyacrylamide gels containing 7M urea at a constant wattage of 50 W, and visualized by phosphorimaging.

For the test of guide RNA paired with a 12 nt passenger strand fragment, 1 µM of phosphorylated guide RNA was mixed with 2 µM of phosphorylated 12 nt passenger fragment in 50 µL of annealing buffer [100 mM Potassium acetate, 25 mM HEPES-KOH (pH 7.4), 5 mM Magnesium acetate, and 1 unit/μL RiboLock RNase Inhibitor (Thermo Fisher)] in a microcentrifuge tube. Annealing was achieved by heating the oligonucleotide mixture in a boiling water bath and then allowing the water bath to cool to room temperature. Recombinant AGO4 (approximately 1 µg) was incubated with annealed RNAs containing 40 nM guide-strand and 80 nM 12 nt fragment in 20 µL of reaction buffer containing 1 unit/μL RiboLock RNase Inhibitor (Thermo Fisher) for 1 hour at room temperature to allow RNA incorporation into AGO4. 1 µL ^32^P-phosphorylated target RNA (~1000 cpm/µL) and 1 µg yeast tRNA (Thermo Fisher Scientific) was then added to each reaction. After 1-hour incubation at room temperature, reactions were quenched by adding 200 µL of stop solution (300 mM NaOAc and 7M urea). RNAs were extracted using 200 µL of phenol/chloroform/isoamyl alcohol, ethanol precipitated, resuspended in 1X formamide loading buffer, resolved in 15% polyacrylamide gels containing 7M urea at a constant wattage of 50 W, and visualized by phosphorimaging.

***In vitro* guide RNA loading assay**

To test if guide siRNAs with 5’ A, U, G, and C can be incorporated into AGO4 (Figure 4D), recombinant AGO4 (approximately 1 µg) was incubated with 10 nM ^32^P-phosphorylated guide RNAs in 20 µL of reaction buffer containing 1 unit/μL RiboLock RNase Inhibitor (Thermo Fisher) for 1 hour at room temperature to allow guide RNA loading. AGO4 proteins were then subjected to immunoprecipitation using 8 µL of anti-FLAG antibody-conjugated Dynabeads. Following 1 hour of incubation at room temperature, the beads were magnet-precipitated and washed three times with 0.5 mL wash buffer [600 mM Potassium acetate, 25 mM HEPES-KOH (pH 7.4), 5 mM Magnesium acetate, 10% glycerol, 0.1% IGEPAL, 0.5 mM DTT, and 1 mM PMSF]. Reactions were quenched by adding 200 µL of stop solution (300 mM NaOAc and 7M urea). RNA was extracted by 200 µL phenol/chloroform/isoamyl alcohol, ethanol precipitated, resuspended in 1x formamide loading buffer, and subject to electrophoresis in 15% polyacrylamide gels containing 7M urea at a constant wattage of 50 W. The gels were dried and visualized by phosphorimaging. For competition assays, cold phosphorylated guide RNA was mixed with ^32^P-phosphorylated guide RNA that contains a 5’ adenosine. The mixed RNA was then subjected to AGO4 loading, immunoprecipitation, and electrophoresis as described above.

**RNA blot analysis**

Recombinant AGO4 or AGO4-SD (approximately 1 µg each) was incubated with 40 nM of guide siRNA, having either 5’ monophosphate, 5’ hydroxyl, or 5’ triphosphate groups, in 20 µL of reaction buffer containing 1 unit/μL RiboLock RNase Inhibitor (Thermo Fisher) for 1 hour at room temperature. AGO4-siRNA complexes were immunoprecipitated by incubating with 8 µL of FLAG antibody-conjugated Dynabeads for 2 hours at 4 ˚C. The beads were then washed twice with 0.5 mL of wash buffer. Reactions were quenched by adding 200 µL of stop solution containing 300 mM NaOAc and 7M Urea to the beads. RNAs were extracted by 200 µL phenol/chloroform/isoamyl alcohol, ethanol precipitated, resuspended in 1X formamide loading buffer, and resolved in a 15% polyacrylamide gel containing 7M urea. RNAs were then transferred to a nylon membrane (Amersham Hybond N+) using 0.5X TBE buffer in a semi-dry blotter (Bio-Rad) at constant voltage of 20 V for 45 minutes. The RNA was then chemically crosslinked to the membrane with 31 mg/mL of 1-ethyl-3-(3-dimethylaminopropyl)-carbodiimide (EDC) for 2 hours at 60 ˚C. The membrane was washed 5 times with water, followed by prehybridization using PerfectHyb Plus buffer (Sigma) containing 100 µg /mL sheared salmon sperm DNA (Ambion) at 42 ˚C for 1 hour. The membrane was then hybridized to 1 µM 5’-end ^32^P-labeled DNA probe overnight in PerfectHyb Plus buffer containing 100 µg /mL sheared salmon sperm DNA at 42 ˚C. Following hybridization, the membrane was washed twice with non-stringent RNA blot wash buffer [3x SSC, 25 mM NaH_2_PO_4_ (pH 7.5), and 5% SDS] at 50 ˚C for 10 minutes, twice with non-stringent RNA blot wash buffer at 50 ˚C for 30 minutes, followed by one wash with stringent RNA blot wash buffer (1X SSC and 1% SDS) at 50 ˚C for 5 minutes. The membrane was analyzed by phosphorimaging.
