# Supplementary material for: Enzymatic reactions of AGO4 in RNA-directed DNA methylation: siRNA duplex loading, passenger strand elimination, target RNA slicing, and sliced target retention
Source: Genes Dev. 2023 Feb 1;37(3-4):103–18. doi: 10.1101/gad.350240.122 (PMC10069450; doi:10.1101/gad.350240.122)
Supplement: Supplemental Material [file supp_gad.350240.122_Supplemental_Figure_Legends.docx]

**Supplemental Figure Legends**

**Supplemental Figure S1. Relative abundance of 23 and 24 nt RNAs associated with AGO4 or in the total small RNA pool. Related to Figures 2A and 2B.**

**A.** AGO4 and AGO4-SD protein levels in transgenic *A. thaliana* lines. For each line,100 mg of 10-day-old seedling tissue was ground into fine powder in liquid nitrogen then resuspended in 200 μL of 2X SDS-PAGE sample buffer. 30 μL of each sample was then loaded onto a 4-20% SDS-PAGE gel, resolved by electrophoresis and subjected to immunoblotting. Anti-FLAG antibody (A8592) was used to detect FLAG-tagged AGO4 or AGO4-SD proteins. Histone H3 proteins were detected using anti-Histone H3 antibody (ab21054).

**B.** Relative abundance of 23 nt and 24 nt siRNAs co-immunoprecipitated with wild-type AGO4 or slicing-defective AGO4-SD at loci at which 24 nt siRNAs predominate. Boxplots show medians (horizontal lines), 1st–3rd quartile range (boxes), other data extending to 1.5 times the interquartile range (whiskers), and outliers for two independent replicates.

**C**. Relative abundance of 23 nt and 24 nt siRNAs in the pool of total RNAs purified from inflorescence tissues. Total RNAs were extracted from stable transgenic lines expressing wild-type or slicing-defective AGO4 in the *ago4-4* null mutant background. Results of three independent replicates are shown.

**Supplemental Figure S2. Sequence features of AGO4-associated small RNAs. Related to Figures 2A and 2B.**

**A**. Sequence logos for 22-26 nt small RNAs that co-immunoprecipitated with wild-type AGO4.

**B**. Sequence logos for 22-26 nt small RNAs that co-immunoprecipitated with slicing-defective AGO4-SD.

**C**. Genome mismatch frequency for each nucleotide position of 22-26 nt small RNAs that co-immunoprecipitated (IPed) with wild-type AGO4 or slicing-defective AGO4-SD or the mock IP control.

**Supplemental Figure S3. Computational reconstruction of 24/23 or 24/24 siRNA duplexes associated with slicing-defective AGO4. Related to Figures 2C and 2D.**

**A**. Computational strategy for prediction of AGO4-bound siRNA duplexes. RNAs associated with slicing-defective AGO4, thus representing both guide and passenger strands, were tested for optimal basepairing alignments. For 24 nt and 23 nt siRNAs that overlap with at least 1 nucleotide of complementarity, the 5’ to 3’ registry is defined as the distance from the 5’ nucleotide of the top strand to the 3’ nucleotide of the bottom strand. The frequency of every possible 5’ to 3’ registry in the population of all observed 24/23 nt pairs was calculated.

**B**. Predicted pairing frequencies represented by the possible 5’ to 3’ registries for 24/23 nt siRNA pairs. The most highly represented pairing pattern is illustrated at the bottom of the panel.

**C**. Predicted pairing frequencies represented by the possible 5’ to 3’ registries for 24/24 nt siRNA pairs in AGO4-SD and wild-type AGO4. The most highly represented pairing pattern for 24 nt RNAs copurifying with slicing-defective AGO4 is illustrated at the bottom of the panel.

**Supplemental Figure S4. Comparison of the human AGO2 structure to the predicted structures for *A. thaliana* AGO1 and AGO4 in the region near the guide strand RNA’s 3’ end. Related to Figure 3C.**

A. Crystal structure for human AGO2 in association with miR20 (PDB: 4F3T) and AlphaFold-predicted structures for *A. thaliana* AGO1 and AGO4. An extended β hairpin and positively charged disordered loop in the predicted structure of AGO4 is highlighted.

B. Sequence comparisons of predicted motifs predicted to be in proximity to guide strand RNA 3’ ends are shown for the ten A*. thaliana* AGO proteins and human AGO2. Residues are color-coded based on degree of conservation, with warmer colors representing higher conservation.

**Supplemental Figure S5. Comparison of the AGO1 phosphate binding pocket for the guide strand’s 5’ end to the equivalent structure predicted for AGO4. Related to Figure 4B and 4C.**

A. The MID domain of *Arabidopsis* AGO4 predicted using AlphaFold is superimposed onto the crystal structure for the *Arabidopsis* AGO1 MID domain in complex with the phosphate group of Adenosine monophosphate (PDB: 4G0Y). Residues in AGO1 predicted to contact the 5’ phosphate are highlighted. Predicted hydrogen bonds are denoted by dashed lines. The nucleotide specificity loop is denoted by a dashed circle. The numbering of highlighted amino acids is based on the *Arabidopsis* AGO1 sequence.

B. Sequence comparison of the predicted phosphate-binding pocket in the ten *A. thaliana* AGO proteins and human AGO2. Key residues predicted to contact the 5’ terminal phosphate are highlighted. Residues are color-coded based on conservation, with warmer color represents more highly conserved sequences.

**Supplemental Figure S6. Competition assays testing whether the 5’ terminal nucleotide of 24 nt guide RNAs affects AGO4 binding. Related to Figures 4D and 4E.**

FLAG-tagged AGO4 binding to a ^32^P end-labeled 24 nt siRNA beginning with a 5’ A was carried out in the presence of increasing concentrations of unlabeled phosphorylated 24 nt siRNAs that begin with 5’ A, U, G, or C but are otherwise identical in sequence. AGO4 was then affinity captured and associated RNAs were resolved by denaturing PAGE and autoradiography.

**Supplemental Figure S7. Influence of AGO4 slicing activity on CHH, CHG, and CG methylation at transposable elements**

Arabidopsis short TEs (1-2 kb) and long TEs (>4kb) that overlap with AGO4-dependent DMRs were aligned at their 5’ and 3’ ends. Average cytosine methylation for all cytosines within 50 bp intervals in TE bodies and 2 kb upstream and downstream flanking regions is plotted.

**Supplemental Table S1. Genomic positions for AGO4-associated RNA clusters**

**Supplemental Table S2. CHH methylation ratios at AGO4 Differentially Methylated Regions (DMRs)**

**Supplemental Table S3. Oligonucleotides used in the study**
