# Supplementary material for: Enzymatic reactions of AGO4 in RNA-directed DNA methylation: siRNA duplex loading, passenger strand elimination, target RNA slicing, and sliced target retention
Source: Genes Dev. 2023 Feb 1;37(3-4):103–18. doi: 10.1101/gad.350240.122 (PMC10069450; doi:10.1101/gad.350240.122)

A. AGO4 and AGO4-SD protein levels in transgenic *A. thaliana* lines

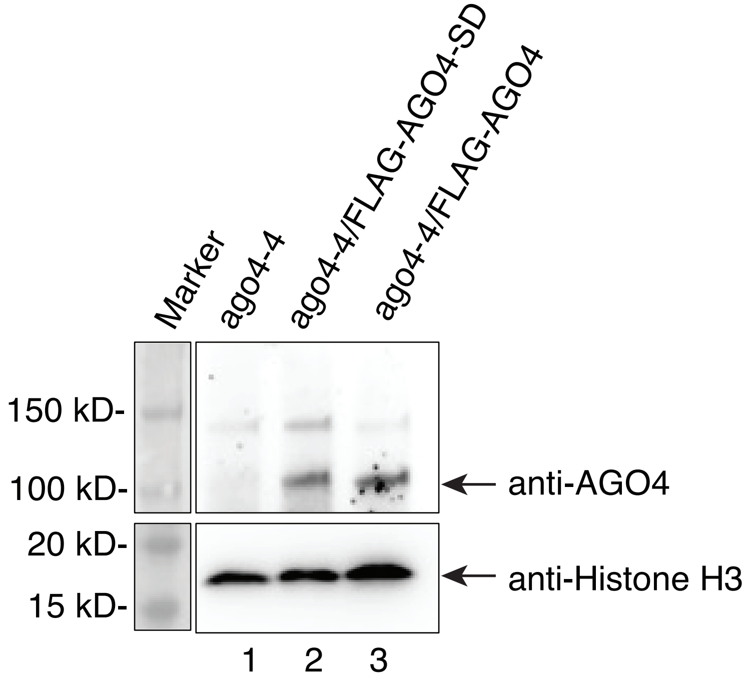

B. Relative abundance of AGO4-associated small RNAs

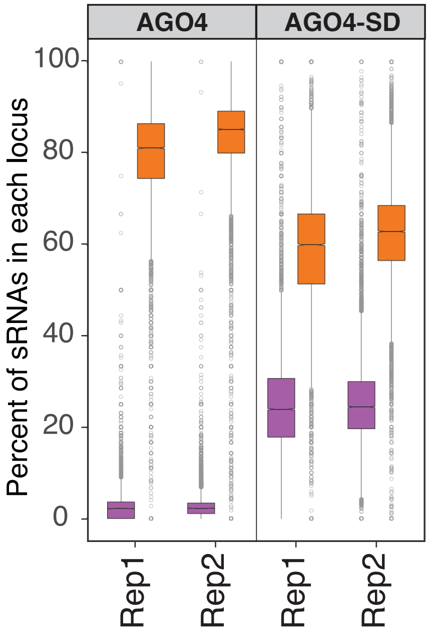

C. Relative abundance of total small RNAs

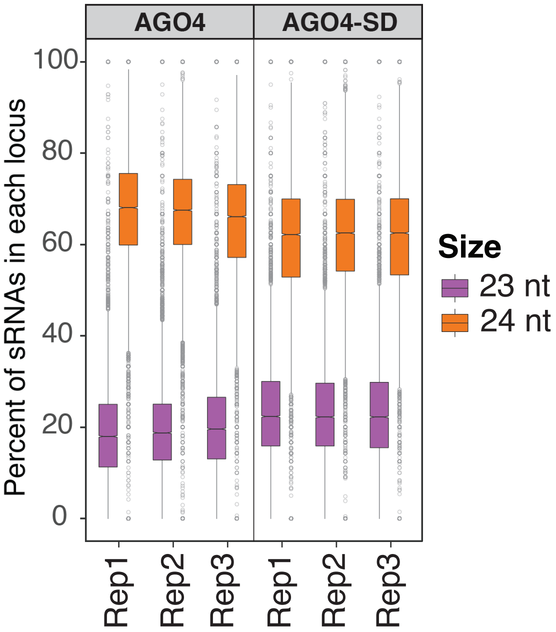

Supplement: Supplemental Material [file supp_gad.350240.122_Supplemental_FigS1.pdf]
