# Supplementary material for: Enzymatic reactions of AGO4 in RNA-directed DNA methylation: siRNA duplex loading, passenger strand elimination, target RNA slicing, and sliced target retention
Source: Genes Dev. 2023 Feb 1;37(3-4):103–18. doi: 10.1101/gad.350240.122 (PMC10069450; doi:10.1101/gad.350240.122)
Supplement: Supplemental Material [file supp_gad.350240.122_Supplemental_FigS3.pdf]

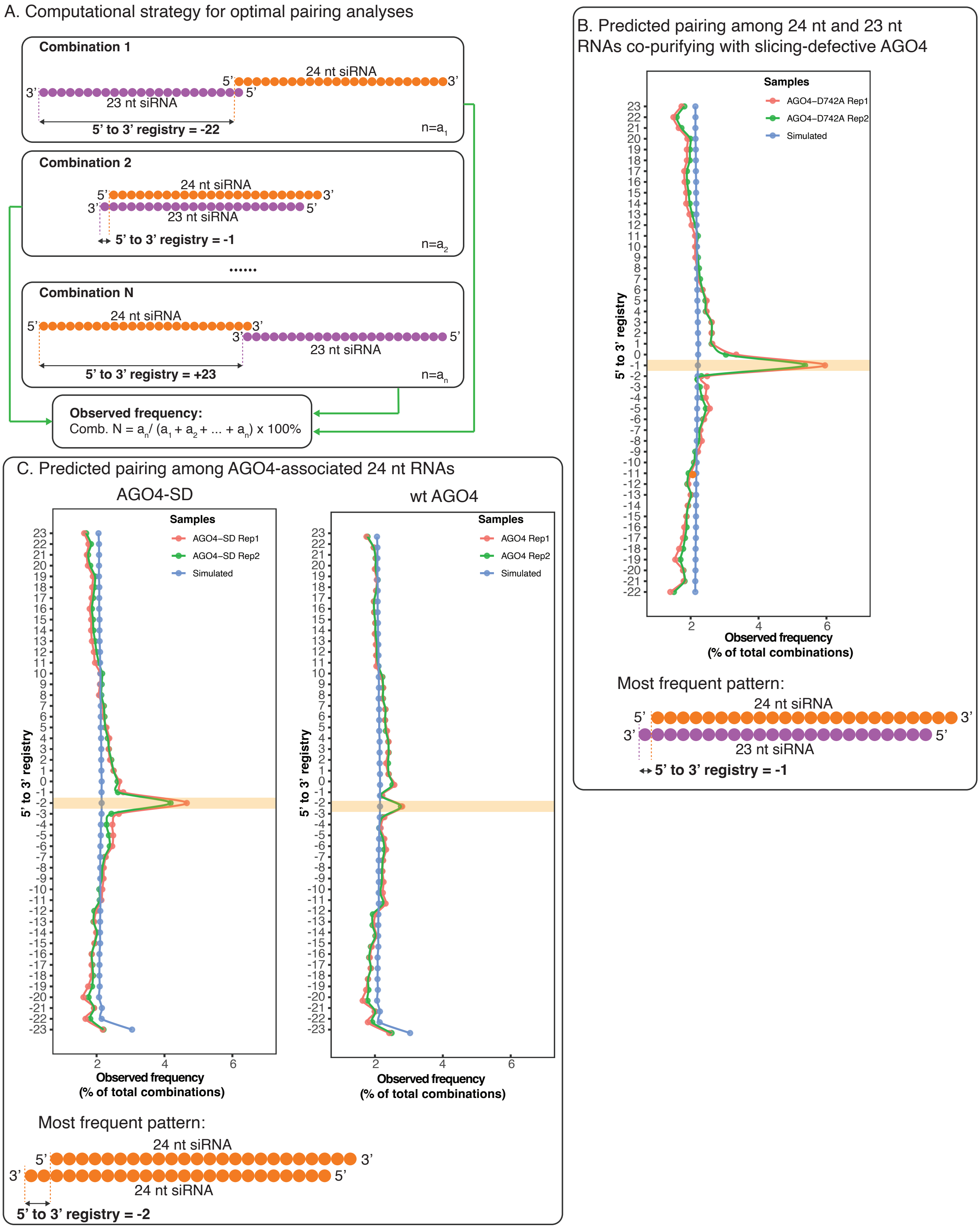
A. Computational strategy for optimal pairing analyses
Combination 1
24 nt siRNA
23 nt siRNA
5' to 3' registry = -22
n=a1
Combination 2
24 nt siRNA
23 nt siRNA
5' to 3' registry = -1
n=a2
Combination N
24 nt siRNA
23 nt siRNA
5' to 3' registry = +23
n=an
Observed frequency:
Comb. N = an / (a1 + a2 + ... + an) x 100%
B. Predicted pairing among 24 nt and 23 nt RNAs co-purifying with slicing-defective AGO4
Samples
AGO4–D742A Rep1
AGO4–D742A Rep2
Simulated
5' to 3' registry
Observed frequency (% of total combinations)
Most frequent pattern:
24 nt siRNA
23 nt siRNA
5' to 3' registry = -1
C. Predicted pairing among AGO4-associated 24 nt RNAs
AGO4-SD
Samples
AGO4–SD Rep1
AGO4–SD Rep2
Simulated
wt AGO4
Samples
AGO4 Rep1
AGO4 Rep2
Simulated
5' to 3' registry
Observed frequency (% of total combinations)
Most frequent pattern:
24 nt siRNA
24 nt siRNA
5' to 3' registry = -2
