# Supplementary material for: Enzymatic reactions of AGO4 in RNA-directed DNA methylation: siRNA duplex loading, passenger strand elimination, target RNA slicing, and sliced target retention
Source: Genes Dev. 2023 Feb 1;37(3-4):103–18. doi: 10.1101/gad.350240.122 (PMC10069450; doi:10.1101/gad.350240.122)

A. The structure of human AGO2 loaded with miR20 determined by crystallography and predicted structures of Arabidopsis AGO1 and AGO4

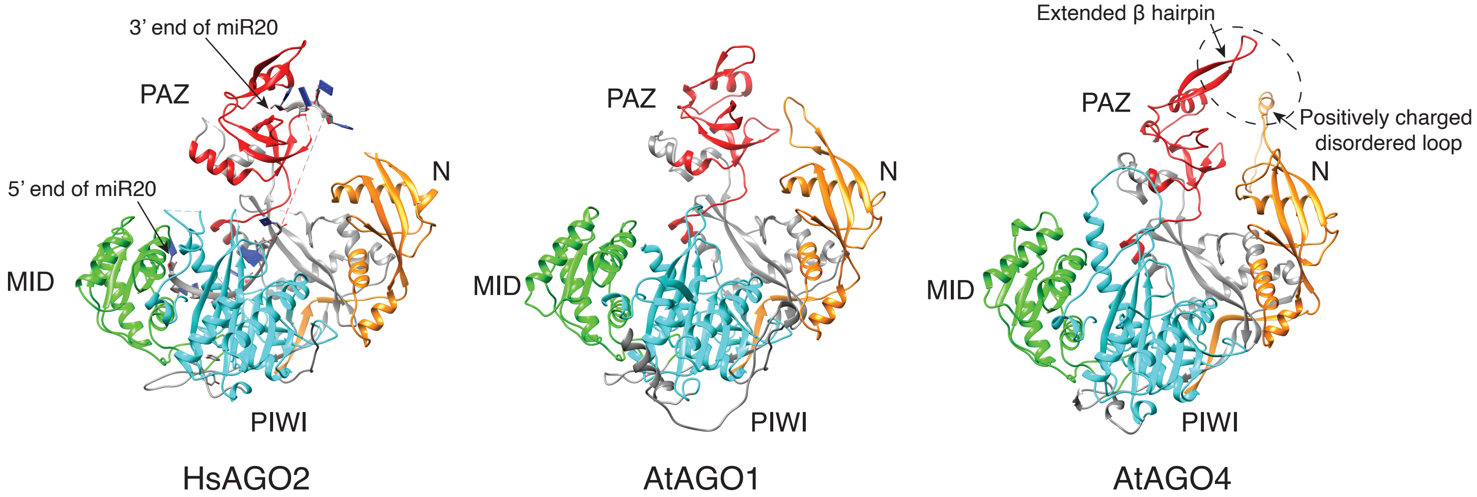

B. Sequence comparison of predicted motifs in proximal to the 3' end of guide-strand RNA

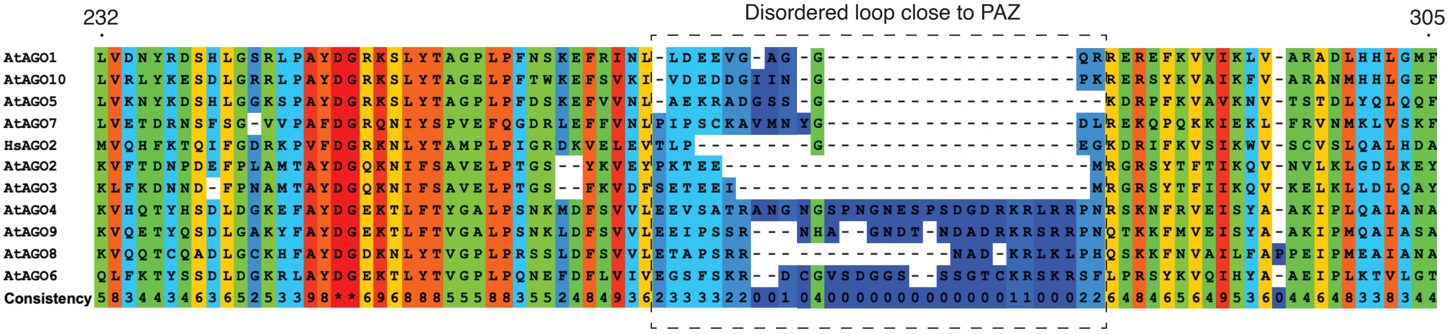

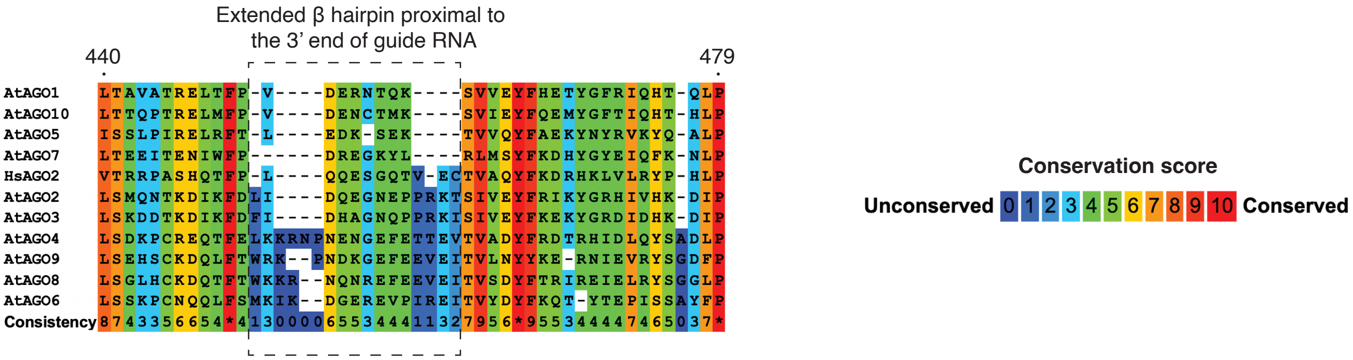

Supplement: Supplemental Material [file supp_gad.350240.122_Supplemental_FigS4.pdf]
