# Supplementary material for: Enzymatic reactions of AGO4 in RNA-directed DNA methylation: siRNA duplex loading, passenger strand elimination, target RNA slicing, and sliced target retention
Source: Genes Dev. 2023 Feb 1;37(3-4):103–18. doi: 10.1101/gad.350240.122 (PMC10069450; doi:10.1101/gad.350240.122)
Supplement: Supplemental Material [file supp_gad.350240.122_Supplemental_FigS5.pdf]

A. Predicted AGO4 MID domain structure superimposed onto the known structure for the AGO1 MID domain binding an AMP molecule

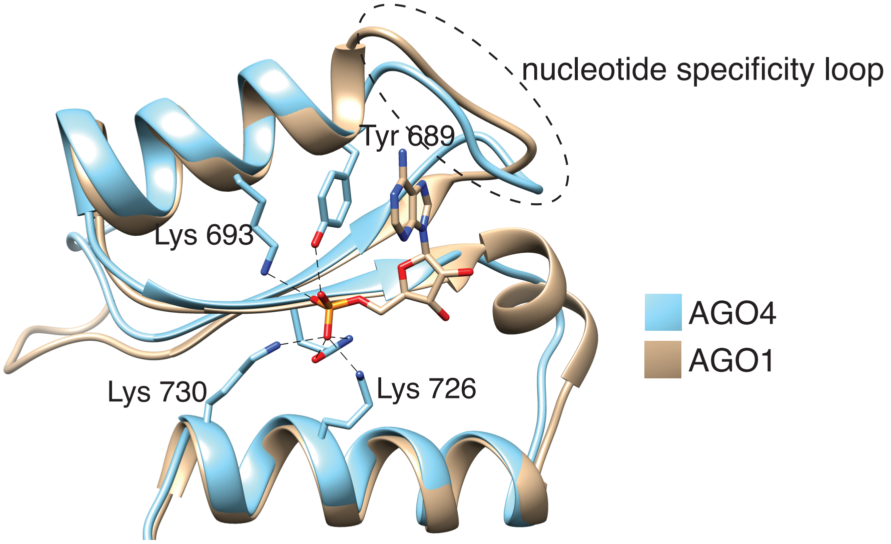

B. Sequence alignments for predicted phosphate-binding pockets of AGO MID domains

(689 and 693 are marked with arrows above the third and fourth blocks.)

AtAGO1	ATS-KLSQGK	EIDLLIVILP	D-NNGSLYGD	LKRICETELG	IVSQCCLTKH
AtAGO10	SMN-K-TKGK	ELELLLAILP	D-NNGSLYGD	LKRICETELG	LISQCCLTKH
AtAGO5	----KRAPG-	-LQLLIVILP	D-VTGS-YGK	IKRICETELG	IVSQCCQPRQ
AtAGO7	LKEIQRAASN	NLQLIICVME	K-KHKG-YGD	LKRISETRIG	VVTQCCLYPN
HsAGO2	------YAG-	-LQLVVVILP	G-KT-PVYAE	VKRVGDTVLG	MATQCVQMKN
AtAGO2	AS--RKHGGA	RPTLVLCAMS	R-KDDG-YKT	LKWIAETKLG	LVTQCFLTGP
AtAGO3	AS--HNHGGA	CPTLVLCAMT	G-KHDG-YKT	LKWIAETKLG	LVTQCFLTIS
AtAGO4	-------LPG-	VPQFILCVLP	DKKNSDLYGP	WKKKNLTEFG	IVTQCMAPTR
AtAGO9	-------LPK-	PPLFLLCILA	ERKNSDVYGP	WKKKNLVDLG	IVTQCIAPTR
AtAGO8	-------LGEV	PPKFLLCIL-	EKKNSDVY--	--EKSCSMWN	--CECIVPPQ
AtAGO6	-------FPD-	PPHFILCILP	ERKTSDIYGP	WKKICLTEEG	IHTQCICPIK
Consistency	1000103451	2447886784	5065453*53	4856448558	6579*44434

(726 and 730 are marked with arrows above the second and third blocks.)

AtAGO1	VFK---MSKQ	YMANVALKIN	VKVGGRNTVL	VDALSRRIP-	-LVSDRPTII
AtAGO10	VFK---ISKQ	YLANVSLKIN	VKMGGRNTVL	VDAISCRIP-	-LVSDIPTII
AtAGO5	VNK---LNKQ	YMENVALKIN	VKTGGRNTVL	NDAIRRNIP-	-LITDRPTII
AtAGO7	ITK---LSSQ	FVSNLALKIN	AKIGGSMTEL	YNSIPSHIPR	LLRPDEPVIF
HsAGO2	VQR---TTPQ	TLSNLCLKIN	VKLGGVNNIL	LP--QGRPP-	--VFQQPVIF
AtAGO2	ATKG---GDQ	YRANLALKMN	AKVGGSNVEL	MDT-FSFFK-	---KEDEVMF
AtAGO3	AIKGETVSDQ	YLANLALKIN	AKVGGTNVEL	VDNIFSFFK-	---KEDKVMF
AtAGO4	Q-----PNDQ	YLTNLLLKIN	AKLGGLNSML	SVERTPAFT-	-VISKVPTII
AtAGO9	L------NDQ	YLTNVLLKIN	AKLGGLNSLL	AMERSPAMP-	-KVTQVPTII
AtAGO8	N-----LNDQ	YLTNLLLKIN	AKLGGLNSVL	DMELSGTMP-	-LVMRVPTII
AtAGO6	I------SDQ	YLTNVLLKIN	SKLGGINSLL	GIEYSYNIP-	-LINKIPTLI
Consistency	5130002 65*	876*75**9*	6*7**4855*	3343433560	0344537787

Conservation score

Unconserved 0 1 2 3 4 5 6 7 8 9 10 **Conserved**
