# Supplementary material for: Enzymatic reactions of AGO4 in RNA-directed DNA methylation: siRNA duplex loading, passenger strand elimination, target RNA slicing, and sliced target retention
Source: Genes Dev. 2023 Feb 1;37(3-4):103–18. doi: 10.1101/gad.350240.122 (PMC10069450; doi:10.1101/gad.350240.122)
Supplement: Supplemental Material [file supp_gad.350240.122_Supplemental_TableS3.pdf]

Table S3. Oligonucleotides used in the study

Name	Sequences (5' to 3')	Where the oligo was used
23 nt target RNA	rGrGrCrArUrGrGrArUrGrArArCrUrArUrArCrArArArUmA	Fig. 3 and 4
5' A 24 nt guide RNA	rArUrUrUrGrUrArUrArGrUrUrCrArUrCrCrArUrGrCrCrAmU	Fig. 3 and 4, Supplemental Fig. 6
5' U 24 nt guide RNA	rUrUrUrUrGrUrArUrArGrUrUrCrArUrCrCrArUrGrCrCrAmU	Fig. 4, Supplemental Fig. 6
5' G 24 nt guide RNA	rGrUrUrUrGrUrArUrArGrUrUrCrArUrCrCrArUrGrCrCrAmU	Fig. 4, Supplemental Fig. 6
5' C 24 nt guide RNA	rCrUrUrUrGrUrArUrArGrUrUrCrArUrCrCrArUrGrCrCrAmU	Fig. 4, Supplemental Fig. 6
24 nt guide RNA with central mismatches	rArUrUrUrGrUrArUrUrCrArUrCrArUrCrCrArUrGrCrCrAmU	Fig. 5
Triphosphorylated 24 nt guide RNA	[PPP]rArUrUrUrGrUrArUrArGrUrUrCrArUrCrCrArUrGrCrCrAmU	Fig. 4
23 nt guide RNA	rArUrUrUrGrUrArUrArGrUrUrCrArUrCrCrArUrGrCrCmA	Fig. 3
22 nt guide RNA	rArUrUrUrGrUrArUrArGrUrUrCrArUrCrCrArUrGrCmC	Fig. 3
21 nt guide RNA	rArUrUrUrGrUrArUrArGrUrUrCrArUrCrCrArUrGmC	Fig. 3
18 nt guide RNA	rArUrUrUrGrUrArUrArGrUrUrCrArUrCrCmA	Fig. 3
16 nt guide RNA	rArUrUrUrGrUrArUrArGrUrUrCrArUmC	Fig. 3
14 nt guide RNA	rArUrUrUrGrUrArUrArGrUrUrCmA	Fig. 3
Synthesized 12 nt marker RNA	rGrGrCrArUrGrGrArUrGrArA	Fig. 3 and 4
51 nt target RNA	rCrGrArGrUrCrGrGrArCrCrArGrGrCrArUrGrGrCrArUrGrGrArUr GrArArCrUrArUrArCrArArArUrGrArCrUrCrGrArArUrUrArG	Fig. 5
T7 in vitro transcription template DNA to obtain the 54 nt target RNA	CTAATTCGAGTCATTTGTATAGTTCATCCATGCCATGCCTG GTCCGACTCGCCCTATAGTGAGTCGTATTA	Fig. 5
T7 promoter	TAATACGACTCACTATAGGG	Fig. 5
BsaI_AGO4_F	ATGGTCTCAAGGTGACTACAAAGACGACGAC	Forward primer Subcloning AGO4 into SUMOstar vector
XbaI_AGO4_R	GATCTAGATTAACAGAAGAACATACTATT	Reverse primer Subcloning AGO4 into SUMOstar vector
